# Skeletal muscle gene expression in response to resistance exercise: sex specific regulation

**DOI:** 10.1186/1471-2164-11-659

**Published:** 2010-11-24

**Authors:** Dongmei Liu, Maureen A Sartor, Gustavo A Nader, Laurie Gutmann, Mary K Treutelaar, Emidio E Pistilli, Heidi B IglayReger, Charles F Burant, Eric P Hoffman, Paul M Gordon

**Affiliations:** 1 Laboratory for Physical Activity and Exercise Intervention Research, Dept. of Physical Medicine and Rehabilitation, University of Michigan, Ann Arbor, MI, USA; 2 Center for Computational Medicine and Bioinformatics, University of Michigan, Ann Arbor, MI, USA; 3 Department of Medicine, Karolinski Institute, Stockholm, Sweden; 4 Department of Neurology, West Virginia University, Morgantown, WV, USA; 5 Department of Internal Medicine, University of Michigan, Ann Arbor, MI, USA; 6 Department of Physiology and Pennsylvania Muscle Institute, University of Pennsylvania, Philadelphia, PA, USA; 7 Research Center for Genetic Medicine, Children's National Medical Center, Washington, DC, USA

## Abstract

**Background:**

The molecular mechanisms underlying the sex differences in human muscle morphology and function remain to be elucidated. The sex differences in the skeletal muscle transcriptome in both the resting state and following anabolic stimuli, such as resistance exercise (RE), might provide insight to the contributors of sexual dimorphism of muscle phenotypes. We used microarrays to profile the transcriptome of the biceps brachii of young men and women who underwent an acute unilateral RE session following 12 weeks of progressive training. Bilateral muscle biopsies were obtained either at an early (4 h post-exercise) or late recovery (24 h post-exercise) time point. Muscle transcription profiles were compared in the resting state between men (n = 6) and women (n = 8), and in response to acute RE in trained exercised vs. untrained non-exercised control muscle for each sex and time point separately (4 h post-exercise, n = 3 males, n = 4 females; 24 h post-exercise, n = 3 males, n = 4 females). A logistic regression-based method (LRpath), following Bayesian moderated t-statistic (IMBT), was used to test gene functional groups and biological pathways enriched with differentially expressed genes.

**Results:**

This investigation identified extensive sex differences present in the muscle transcriptome at baseline and following acute RE. In the resting state, female muscle had a greater transcript abundance of genes involved in fatty acid oxidation and gene transcription/translation processes. After strenuous RE at the same relative intensity, the time course of the transcriptional modulation was sex-dependent. Males experienced prolonged changes while females exhibited a rapid restoration. Most of the biological processes involved in the RE-induced transcriptional regulation were observed in both males and females, but sex specificity was suggested for several signaling pathways including activation of notch signaling and TGF-beta signaling in females. Sex differences in skeletal muscle transcriptional regulation might implicate a mechanism behind disproportional muscle growth in males as compared with female counterparts after RE training at the same relative intensity.

**Conclusions:**

Sex differences exist in skeletal muscle gene transcription both at rest and following acute RE, suggesting that sex is a significant modifier of the transcriptional regulation in skeletal muscle. The findings from the present study provide insight into the molecular mechanisms for sex differences in muscle phenotypes and for muscle transcriptional regulation associated with training adaptations to resistance exercise.

## Background

Sex differences in muscle morphology, function and plasticity have previously been documented. In general, men are stronger and have a larger muscle fiber cross-sectional area, especially for type II fibers [1,2]. In contrast, women generally have a higher proportion of oxidative type I muscle fibers and muscle capillary density [2], and are more resistant to muscle fatigue [3-5]. No influence of sex on strength loss and recovery pattern following damaging eccentric contractions has been reported [6]. Sex differences in skeletal muscle response and adaptation to physiological stimuli, such as training and detraining, have also been reported. Men generally experience a greater hypertrophic response after resistance training in both young [7-10] and older adults [8,11] and also appear to have a higher degree of muscle loss with detraining [10]. Despite the obvious influence of sex on muscle morphology and muscle plasticity, less is known about the molecular events driving the manifestation of sexual dimorphism observed in muscle phenotypes.

Skeletal muscle plasticity in response to exercise is controlled by several levels of regulation, including transcriptional, post-transcriptional and translational events. It has been suggested that the transient changes in transcription during recovery from acute bouts of exercise may accumulate and translate into cellular training adaptations if the exercise is performed for a prolonged period of time [12,13]. Because exercise is a complex stimuli involving mechanical loading, metabolic disturbances, neuronal activation and hormonal alterations [14], microarrays can be a useful high-throughput means to examine global transcriptional profiles and transcriptional changes in skeletal muscle under various experimental conditions. Microarray studies have previously characterized gene expression profiles in human skeletal muscle in relation to sex, age, endurance and resistance training [15,16]. However, no study has examined the global alteration in gene expression profiles following acute resistance exercise (RE) in humans; nor is there much known regarding how men and women differ in RE-induced transcriptional regulation in skeletal muscle.

In this study, we used microarrays to analyze the muscle transcriptome at rest and following acute RE at two time points among young male and female participants. This study was an ancillary study conducted on a subset of participants participating in a larger scale multi-center study, the Functional SNPs Associated with Human Muscle Size and Strength (FAMuSS) study. The FAMuSS study was designed to uncover novel genetic polymorphisms associated with human muscle phenotypes and training adaptation [7,17]. In FAMuSS, a 12 week unilateral arm RE training program was utilized, and the last RE session was repeated 48~96 h after completion of the training as the acute stimuli among 16 volunteers for the present investigation. The aims of the study were 1) to define acute transcriptional regulatory events induced by RE in trained muscle for each sex which potentially mediate skeletal muscle training adaptation; and 2) to identify the sexual differences in the skeletal muscle transcriptome in both the resting untrained state and following RE, as they might contribute to a sexual dimorphism in muscle phenotypes.

Our data analysis focused on identifying significantly regulated biological processes and molecular pathways (i.e., gene functional analysis) instead of testing the differential expression of individual genes. Gene functional analysis has the potential to reduce false positive discoveries typically associated with gene-gene comparison studies [18]. A study conducted by the Texicogenomics Research Consortium [19] clearly demonstrated that in microarray studies the use of gene functional analysis can result in higher reproducibility than gene-gene comparison methods. Moreover, gene-based analysis does not perform as well as gene functional analysis in capturing the minor but concordant changes across multiple genes in a particular pathway, which biologically might be more important than a large fold change in any single gene [20]. Various analytical approaches have been developed and utilized for performing gene functional analysis in microarray studies. Among these is a novel logistic regression-based method (LRpath), which we used in this investigation. LRpath has several documented advantages over many other methods [21]. In particular, it takes into account the distribution of significance levels of all genes profiled and overcomes the limitations associated with the use of arbitrary significance cut-off values [21]. As has been documented, different threshold choices can lead to different results from enrichment analysis, and thus different biological conclusions [22].

## Results

### Physical characteristics of subjects

The subjects' physical characteristics are presented in Table 1. Compared with females, males were heavier, taller and had higher arm flexion strength. The 12-week unilateral arm RE training program induced significant improvements in both muscle mass and strength in the trained arm of the participants. Females, as compared with males, exhibited greater improvements in muscle dynamic strength, measured as one-repetition maximum (Δ1RM%, male vs. female, 42% vs. 54%, p = 0.03). Due to the small number of subjects in the present analysis, the sex difference in training-induced improvements in isometric strength (ΔIso%) and muscle volume (ΔVol%) did not reach significance. However, males tended to have greater improvements in muscle cross-sectional area (CSA) than females (ΔCSA%, male vs. female, 8% vs. 5%, p = 0.08). Nevertheless, we previously reported significantly greater increases in muscle strength in women than in men and a significantly greater increase in muscle CSA in men than in women among 243 male and 342 female participants of the FAMuSS study [7].

**Table 1:** Physical characteristics of subjects.

Variables	Males (n = 8)	Females (n = 8)	p value
Age (yrs)	24.7 ± 0.8	22.7 ± 0.8	ns
Weight (kg)	76.9 ± 5.2	60.0 ± 5.2	0.02
Height (cm)	177.8 ± 3.3	156.2 ± 3.3	< 0.0001
BMI (kg/m2)	24.2 ± 1.7	23.7 ± 1.7	ns
1RM Trained Arm (kg)	16.7 ± 0.8	10.4 ± 0.8	< 0.0001
1RM Control Arm (kg)	12.6 ± 0.5	6.5 ± 0.5	< 0.0001
ISO Trained Arm (kg)	61.5 ± 3.4	29.2 ± 3.4	< 0.0001
ISO Control Arm (kg)	55.4 ± 2.9	25.3 ± 2.9	< 0.0001
CSA Trained Arm (cm2)	6949.3 ± 595.2	5837.1 ± 595.2	ns
CSA Control Arm (cm2)	6314.8 ± 553.3	5544 ± 553.3	ns
VOL Trained Arm (cm3)	642757.5 ± 53086.4	541118.8 ± 53086.4	ns
VOL Control Arm (cm3)	584923.6 ± 50638.3	516896.5 ± 50638.3	ns
Δ1RM relative to control	0.42 (0.29)	0.55 (0.50)	0.03
Δ ISO relative to control	0.10 (0.20)	0.13 (0.07)	ns
Δ CSA relative to control	0.08 (0.05)	0.05 (0.12)	0.08
Δ VOL relative to control	0.10 (0.09)	0.07 (0.10)	ns

Note: Anthropometric and muscle phenotypic measurements are presented as mean ± SEM and sex differences were tested by t-test.

Δ relative to control, relative improvement induced by RE training, calculated as (trained-control)/control; the numbers are median (interquartile range) and sex differences were tested by Wilcoxon Rank Sum Test.

1RM: 1 repetition maximum; ISO: Isometric muscle strength; CSA: Arm whole muscle cross-sectional area measured by MRI; VOL: Arm whole muscle volume.

### Differential gene expression in resting skeletal muscle between men and women

**Figure 1:**
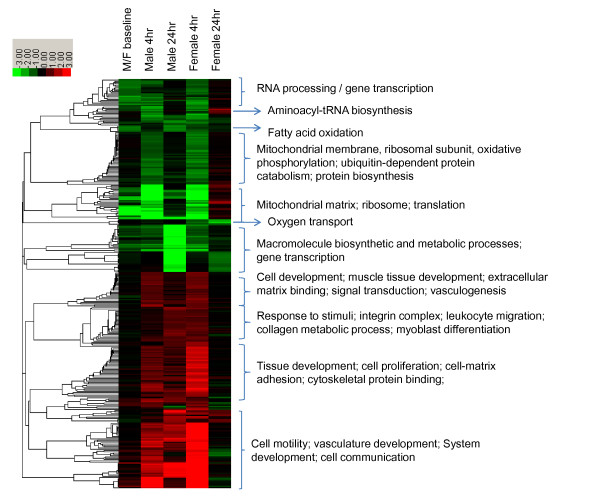
**Clustered GO terms and KEGG pathways significantly enriched for differentially expressed genes in five comparisons**. In total, 318 GO and KEGG pathways with FDR < 0.01 were included in the figure. Each column represents the experimental condition as indicated on the map. Each row represents one GO term/KEGG pathway. These are summarized for each main cluster. Baseline male vs. female (M/F): GO/KEGG significantly enriched for differentially expressed genes in male vs. female muscle in the resting state: Red color indicates strength of enrichment with higher expressed genes in males (or lower expression in females); green indicates enrichment with lower expressed genes in males (or higher expression in females). Male 4 h, Male 24 h, Female 4 h and Female 24 h: GO/KEGG significantly enriched for RE-induced up-and down-regulated genes in male muscle at 4 h (Male 4 h) and 24 h post exercise (Male 24 h), in female muscle at 4 h (female 4 h) and 24 h post exercise (Female 24 h): Red color indicates strength of RE-induced up-regulation and green indicates down-regulation. The -log10(p-value) of enrichment was used as the value for clustering.

We first examined the gene expression profiles of the untrained biceps muscle of male (n = 6) and female (n = 8) participants. Gene functional analysis based on Gene Ontology (GO) terms [23] and Kyoto Encyclopaedia of Genes and Genomes (KEGG) pathways [24] were conducted by using a logistic regression-based method (LRpath [21], see method section). Six GO terms (no KEGG pathway) were significantly enriched for male-associated high expression genes (FDR < 0.01), and 29 GO terms and five KEGG pathways for female-associated high expression genes (FDR < 0.01) (data not shown). After removing highly redundant terms (see methods section), three GO terms were defined as being associated with higher gene expression in male muscle including spermatogenesis (GO:0007283), peptidase activity, acting on L-amino acid peptides (GO:007001) and protein modification by small protein removal (GO:0070646); four GO terms and five KEGG pathways were defined as being associated with higher gene expression in female muscle. These nine functional groups and pathways consistently presented two main biological themes, which were gene transcription and translation and fatty acid oxidation (Figure 1). The complete results of the functional enrichment analysis can be found in supplementary materials (Additional file 1, Table S1).

### Gene transcriptional regulation in skeletal muscle induced by acute resistance exercise

*Men 4 h post (n = 3)*. LRpath analysis following an intensity-based Bayesian moderated t-statistic (IBMT, see the Methods section for details) using a paired sample-design indicated that 29 GO terms (collapsed from 104) and five KEGG pathways were significantly enriched with up-regulated genes; 21GO terms (collapsed from 88) and three KEGG pathways were significantly enriched with down-regulated genes (FDR < 0.01) (Additional file 2, Table S2). The RE up-regulated GO terms and KEGG pathways concentrated on several biological themes including extracellular matrix (ECM) and cytoskeleton based processes, muscle hypertrophy, angiogenesis, signal transduction and stress response; whereas down-regulated GO terms and KEGG pathways were mainly concerned with gene transcription and translation, mitochondrial structure and oxidative phosphorylation activity and protein metabolism (Figure 1).

*Male 24 h post (n = 3)*. In a separate group of males, we discovered in the exercised vs. rested muscle, that 46 GO terms (collapsed from114) and eight KEGG pathways were significantly enriched with up-regulated genes and that 20 GO terms (collapsed from 59) and four KEGG pathways were significantly enriched with down-regulated genes (FDR < 0.01) (Additional file 3, Table S3). Similar to male 4 h post, the biological themes reflected by up-regulated GO terms and KEGG pathways included ECM and cytoskeleton based processes, angiogenesis, signal transduction, muscle hypertrophy and stress response; biological themes reflected by down-regulated GO terms and KEGG pathways concentrated on fatty acid oxidation, carbohydrate and amino acid metabolism, gene transcription and translation, and mitochondrial structure and oxidative phosphorylation activities (Figure 1).

*Female 4 h post (n = 4)*. In exercised vs. rested muscle, up-regulated genes over-represented 17 KEGG pathways and 107 GO terms (collapsed from 320) and down-regulated genes over-represented 36 GO terms (collapsed from 132) and 15 KEGG pathways (Additional file 4, Table S4). The significantly enriched GO terms and KEGG pathways for up-regulated genes can be categorized into several biological functional groups including ECM and cytoskeleton based processes, signal transduction, stress response, tissue regeneration and remodelling, muscle hypertrophy, as well as angiogenesis; whereas down-regulated genes significantly enriched a list of GO terms and KEGG pathways relevant to mitochondrial structure and oxidative phosphorylation activity, proteolysis, and transcription and translation (Figure 1).

*Female 24 h post (n = 4)*. In a separate group of females, we observed in the exercised vs. rested muscle, that seven GO terms (collapsed from 20) and three KEGG pathways were significantly enriched with up-regulated genes and that 13 GO terms and one KEGG pathways with down-regulated genes (Additional file 5, Table S5). Most consistently, the significantly enriched biological GO terms and KEGG pathways for up-regulated genes were related to gene translation and protein biosynthesis (Figure 1).

### Validation of subset of genes by quantitative real time PCR (qRT-PCR)

**Figure 2:**
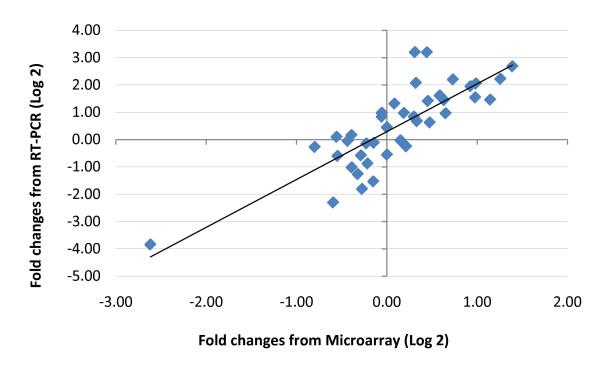
**Correlation between microarray and qRT-PCR on differential expression of ten selected genes**. The blue diamonds represent each gene under four sex and time specific conditions. Spearman's Rho test: Rho = 0.81, p < 0.0001, n = 40.

Ten genes were selected for validation by qRT-PCR. These genes represented seven significantly regulated biological processes by RE, as identified by microarray analysis, including up-regulated growth factor activity (insulin-like growth factor 1, *IGF1; *insulin receptor substrate 2, *IRS2*), angiogenesis (vascular endothelial growth factor A, *VEGFA; *kinase insert domain receptor, *KDR*), stress response (dual specificity phosphatase 1, *DUSP1*), exercise response (peroxisome proliferator-activated receptor gamma, coactivator 1 alpha, *PPARGC1*), down-regulated muscle protein catabolic metabolism (F-box protein 40, *FBXO4; *SMAD family member 3, *SMAD3*), lipid metabolism (aldehyde dehydrogenase 2 family, *ALDH2*), and carbohydrate metabolism (fructose-2,6-biphosphatase 3, *PFKFB3*). These genes were selected based on their known effect in exercise physiology and reported exercise responsiveness. Correlation analysis using Spearman's rank order correlation indicated a strong concordance between fold changes of gene expression tested by microarrays and qRT-PCR in our study (Rho = 0.81, p < 0.0001, n = 40; Figure 2). Because muscle tissue available for qRT-PCR analysis was limited, sample size was not adequate for testing gene expression changes following RE for each of the four sex (male/female) and time (4 h & 24 h post exercise) specific conditions (n = 2, for each of the four groups). As such, we compared mRNA levels of the selected genes in male and female muscles in the resting state (n = 4 for both male and female groups). Consistent with microarray analysis, no significant sex differences were indicated for these genes with the exception of ALDH2. In both the microarray and qRT-PCR, *ALDH2 *showed higher expression levels in females than in males (microarray, fold = 1.38, p = 0.035; qRT-PCR, fold = 2, p = 0.02). The mean fold changes of the expression of these genes in exercised vs. rested muscle for each sex and time specific condition are presented in supplementary materials (Additional file 6, Table S6).

## Discussion

In the present study, we used a novel analytic design to identify sex differences in the human muscle transcriptome in both the resting state and during recovery from acute resistance exercise (RE). In the resting state, female muscle had a greater transcript abundance of genes involved in fatty acid oxidation and gene transcription/translation processes. After strenuous RE at the same relative intensity, transcriptional modulation follows a different time course in male and female muscles (see detailed discussion below). Males experienced prolonged changes while females exhibited a rapid recovery. Moreover, the present study identified novel sex specific areas that exist in the fine regulatory system of the muscle response to RE at the transcriptional level which merit further exploration. The depression of negative regulators of the mTOR signaling pathway in male muscle and activation of notch signaling and TGF-beta signaling in females suggests that sex differences in skeletal muscle transcriptional regulation might implicate a mechanism behind disproportional muscle growth in males as compared with female counterparts after RE training at the same relative intensity. Overall, our data suggest that RE is a powerful modulator of muscle transcriptional regulation that is also tailored by sex.

There are several strengths to the study design used in this investigation. First, we chose to study two time points, i.e 4 h and 24 h post exercise, to address the dynamic and transient nature of gene transcriptional regulation. This choice was based on previous work on the time course of transcriptional changes following exercise [25,26], and represents early and late recovery to acute RE respectively. Secondly, we used separate groups of subjects for each time point. This design has dual advantages: 1) It avoids a second incision to subjects and thus minimizes any possible influence from previous sampling trauma; and 2) Using separate groups of subjects increases the generalizability of the findings and thus improves the validity of our data. Third, to reduce potential confounders, we chose to study biceps brachii instead of the more commonly used quadriceps because 1) sex differences in muscle phenotypes is greater for upper as compared to lower limb musculature[1]; 2) lifestyle influences on upper body are relatively small since lower body musculature plays a dominant role in mobility; 3) compared with vastus lateralis muscle, biceps brachii has a more uniform fiber type distribution between sexes [1]. Given these considerations, studying biceps brachii offers us more power to decipher the sex influence on the muscle transcriptome. However, caution needs to be taken when trying to generalize the present study findings based on biceps brachii to other muscle groups of different fiber type composition. As indicated by a previous study [27], tissue heterogeneity can be a major source of variation in expression profiling experiments even when using relatively homogeneous muscle tissue. Finally, our study design provided the opportunity to maximize analytic power by minimizing the inter-individual biological variability typically observed in human studies. Specifically, subjects were carefully selected: They were of similar age, BMI, and ethnicity, and moreover they were trained under the same exercise protocol. More importantly, we employed a unilateral exercise model with bilateral muscle biopsy procedure, which allowed us to perform a paired comparison within the same individual thus effectively reducing inter-individual variation by controlling all systemic effects on gene expression such as the influence of different genetic backgrounds, neurohormonal states, and other physiological and environmental factors that may exist among individuals.

Furthermore, we studied acute transcriptional regulation of resistance exercise in trained muscle rather than untrained muscle. This approach may have limited our ability to distinguish chronic training effects from acute responses. However this is unlikely to impair the ability of the study to reveal exercise-induced transcriptional regulation relevant to muscle adaptation and, in particular, to affect the detection of sex differences in this regard. Relevant to RE, Tang et al. [28] observed that after eight weeks of unilateral resistance exercise, a shortened, but augmented increase in mixed muscle protein synthesis was shown in the trained leg as compared with untrained control leg in the fed state in young men. Wilkinson et al. [29] compared muscle protein synthetic responses to RE and endurance exercise (EE) before and after ten weeks of training. After training, muscle protein synthetic response became more specific to the training phenotypic adaptation. Resistance exercise increased both mitochondrial and myofibrillar protein synthesis in the untrained state whereas only myofibrillar protein synthesis increased in the trained state [29]. Therefore the design in the present study might provide a better opportunity to observe the mRNA changes more specific to typical resistance exercise training adaptations.

### Sex difference in muscle transcriptome in the untrained resting state

It is plausible to speculate that sex-related gene transcriptional regulation contributes to the sexual dimorphism observed in the skeletal muscle phenotypes [16]. The existence of sex differences in the human skeletal muscle transcriptome has been identified before using genome-wide expression profiling [15,16,30]. Consistent with a recent report [30], females in the present study had higher expression levels of genes involved in lipid metabolism, including lipoprotein lipase (*LPL*), CD36 molecule (*FAT/CD36*), fatty acid binding protein 3 (*FABP3*), hydroxyacyl-Coenzyme A dehydrogenase, beta subunit (*HADHB*) and acyl-Coenzyme A dehydrogenase, long chain (*ACADL*). This finding supports the notion that female muscles rely more on fatty acids as an energy source in the resting state and possibly during submaximal endurance exercise, as demonstrated elsewhere [2]. Kiens et al. [31] observed significantly higher mRNA levels of several key lipid-binding proteins and *LPL *in untrained female vastus lateralis muscle than in male muscle, although a similar trend was not observed for corresponding protein levels. A possible contributor to the sex difference in substrate utilization for energy production in skeletal muscle is estrogen. In a recent study, Fu et al. elegantly demonstrates that female subjects had higher mRNA levels of genes involved in lipid metabolism than male [32] subjects both before and immediately after 90 minutes of cycling (@ 65% VO_2 _peak) and that 17β-estradiol (E2) supplementation among male participants was able to increase the transcript abundance of those lipid metabolism related genes.

Considering that males have larger muscle fibers, we expected to see higher mRNA levels of genes involved in muscle protein biosynthesis in the male and/or higher expression levels of genes involved in protein catabolic processes in the female. Instead, our investigation revealed that the genes involved in transcription and post-transcriptional RNA processing, ribosome construction and mRNA translation were consistently expressed at higher levels in female biceps. This observation suggests that females, at least at the transcriptional level, have a greater potential for protein biosynthesis due to a higher efficiency of gene transcription and translation machinery. Indeed, a recent study [33] reported that women had higher rates of whole-body protein turnover and skeletal muscle protein synthesis than men at both young and old age. The higher protein turnover rates may underlie the need for increased levels of translational machinery.

In comparison to females, males in the rested state demonstrated higher expression levels of genes which enriched GO terms relevant to protein modification by small protein removal. The key genes driving the enrichment of these terms included several ubiquitin specific peptidases. Since ubiquitin conjugation for targeted protein degradation via the proteasome system plays a crucial role in muscle protein proteolysis [34], it is reasonable to postulate that ubiquitin removal (deubiquitination) might outweigh ubiquitination in male muscle thus aiding in muscle protein preservation. However, it should be pointed out that the enrichment of deubiquitination relevant GO terms and KEGG pathways in male muscle may be simply a result of including the Y chromosome in the analysis considering the most significant gene, ubiquitin specific peptidase 9 (*USP9Y*), is Y-linked. Since the biological function of *USP9Y *in skeletal muscle is still unclear, we can only speculate that interference of deubiquitination factors in the protein catabolic process might play a role in protein accumulation in males.

### Sex alters the time course of gene transcriptional regulation after RE

In the present study, we observed that RE induced an extensive alteration in skeletal muscle transcriptome throughout the 24 h recovery period in both male and female muscles. When examining the GO terms and KEGG pathways enriched with differentially expressed genes across the four conditions (Figure 1), we recognized a striking difference between the sexes in the time course of RE-induced transcriptome alteration. Male muscles responded to the exercise stimuli with prolonged alterations in transcript contents, where most of the GO terms and KEGG pathways that were significantly enriched at 4 h post exercise also remained significant at 24 h post exercise. In contrast, female muscle experienced a quick restoration to the baseline state, where nearly all of the significantly enriched GO terms and KEGG pathways were only seen at 4 h post exercise. Additionally, up-regulation of biological processes related to mitochondria ribosome and gene translation were observed only at 24 h post exercise in females. This may suggest that 24 h post RE represents another phase of transcriptional response after recovery from acute exercise in female skeletal muscle. The sex differences in the time course of muscle transcriptional alteration as a result of RE were also confirmed by checking a subset of genes with well established function in RE, i.e., myogenin (*MYOG*), insulin-like growth factor 1 (*IGFI*), nuclear receptor subfamily 4, group A, member (3Nr4a3), ankyrin repeat domain 1 (cardiac muscle) (*ANKRD1*), vascular endothelial growth factor A (*VEGFA*), and eukaryotic translation initiation factor 4E binding protein 1 (*EIF4EBP1*) [35]. In males, nearly all of these genes showed significant up-regulation at both 4 h and 24 h post exercise, but in females these genes only appeared significantly up-regulated at 4 h post exercise (Additional file 7, Table S7). Furthermore, when we examined the number of differentially expressed genes as a result of RE based on a stringent significance level of FDR≤0.05, we found that no gene appeared significantly altered for males at 4 h post-exercise or for females at 24 h post-exercise. However for females at 4 h post-exercise and males at 24 h post-exercise, 1436 probes (representing 1071 unique annotated genes) and 2005 probes (representing 1398 unique annotated genes) were significant, respectively. These results indicate that transcriptional changes in response to acute RE in male muscle were delayed and they peaked at a later time point, as compared with female muscle.

There is limited information regarding the time course of skeletal muscle transcriptional modification as a result of RE, particularly considering sex. Several studies have used multiple (> = 2) time points to investigate the expression of selected genes during recovery following acute resistance exercise, using combined sex groups [26,36] or men only groups [25,37], thus they provided no insight regarding a sex specific time course of expression changes. The novel finding from this study strongly indicates that sex has a fundamental role in determining the time course of gene transcriptional response to RE. It should also be noted that the relative resistance load was identical in both sexes such that differences cannot be explained by the amount of mechanical work performed. Overall, in the present study, females appeared to possess a higher capacity to restore cellular homeostasis after RE stimuli at the transcriptional level. This finding is consistent with the well-documented phenomenon in which, when compared with males, females have a higher capability to maintain cellular homeostasis [4,5]. It is also plausible to propose that the prolonged gene expression response experienced by males might contribute to the more pronounced hypertrophy seen in male muscle after training.

### Common biological processes transcriptionally regulated by RE

Most of the enriched biological GO terms and KEGG pathways for both up- and down-regulated genes observed in our study were shared in males and females. A complete discussion on the common biological processes can be found in the supplemental material (Additional file 8). Briefly, we observed the up-regulation of extracellular matrix (ECM) and actin cytoskeleton, tissue remodelling, angiogenesis, signal transduction, stress response and immune activation in both males and females. Down-regulated biological processes included mitochondrial structure and oxidative phosphorylation, muscle protein proteolysis and biosynthesis. It is important to point out that involvement of these pathways in muscle in response to RE has been previously reported in studies using various methods including protein assays [38,39], custom DNA microarrays [37], RT-PCR [36,39,40], and Northern blotting [41] (see detailed discussion in Additional file 8). The consistency of our results with these previous reports provides additional confirmation of the reliability of our results. Overall, our data supports the notion that the skeletal muscle response to RE is orchestrated by a series of gene regulatory events [42]. It involves not only muscle fibers, but additional supporting structures and cell types comprising the muscle tissue are also influenced, such as the ECM and actin cytoskeleton, blood vessels and neurons. Our expression data also provides supporting evidence for the inhibitory effect of RE on muscle mitochondria functional activity. Regarding muscle hypertrophy, our data suggested that RE-induced muscle protein accretion may occur at the transcriptional level through a decline of protein degradation and stabilization of mRNA at an early time point, and augmented translation at a later time point.

### Sex-specific gene transcriptional regulation in skeletal muscle induced by acute resistance exercise

Although a majority of the biological processes transcriptionally regulated following RE were shared between men and women, some biological processes appeared to be sex specific. In females, we detected up-regulation of genes involved in: Blood coagulation; insulin receptor binding; transforming growth factor beta (TGF-beta) signaling; SMAD binding; Janus kinase (JAK)/signal transducers and activators of transcription (STAT) signaling; and Notch signaling. In males only, we detected an up-regulation of genes involved in: Ion transport; apoptosis/cell death; and P53 signaling pathway. Additionally, several down-regulated GO terms and KEGG pathways were only observed in males including: Triglyceride biosynthetic process; vitamin D receptor binding; and water transporter activity at 24 h post exercise.

**Figure 3:**
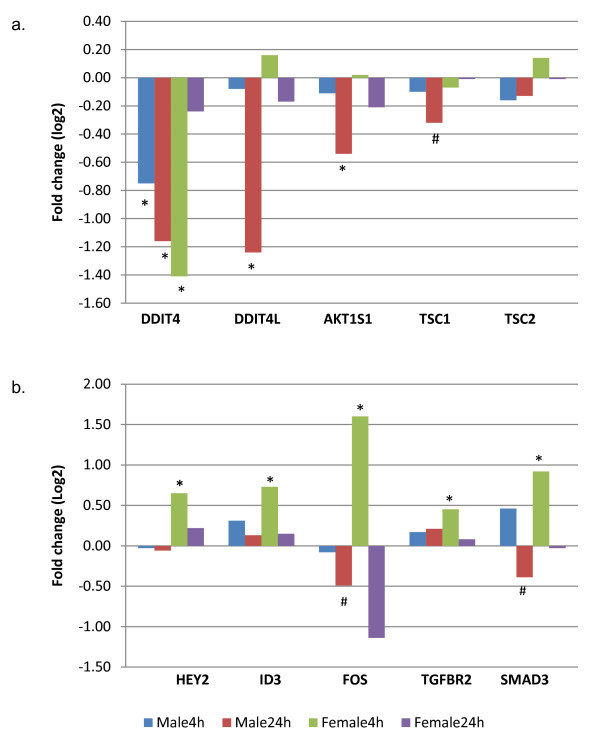
**Sex specific regulation of muscle gene transcription induced by resistance exercise**. a. Repression of negative regulators of mTOR signaling in male muscle. b. Up-regulation of TGF-beta signaling in female muscle. Y axis represents fold changes (log2): Positive values indicate up-regulation post exercise in exercised vs. rested muscle, and negative values indicate down-regulation. * Significantly different from the rested muscle (p < 0.005); # trend towards a differential expression in exercised vs. rested muscle (p < 0.05).

Some of these sex specific features might be a reflection of sex differences in the time course, such that genes whose peak and trough times are out of phase with our sampling time points for one sex but not the other such that they would be detected as specific features for only one sex. Also the imbalance in sample size may skew differential gene expression between sexes, although LRpath has been shown to perform well for small sample sizes [21]. Nevertheless, we cannot exclude the possibility that some of the sex- specific biological processes regulated by RE, defined by this study, have a biological basis. Specifically, coordinated up-regulation of genes related to SMAD binding, TGF-beta signaling and notch signaling may suggest a mechanism for a hampered hypertrophic effect of RE in females (Figure 3). These genes included SMAD family member 1, 3 and 7 (*SMAD1, SMAD3, SMAD7*), transforming growth factor, beta receptor II (*TGFBR2*), v-fos FBJ murine osteosarcoma viral oncogene homolog (*FOS*), interleukin 6 receptor (*IL6R*), hairy/enhancer-of-split related with YRPW motif 1, 2 and motif-like (*HEY1, HEY2, HEYL*), hairy and enhancer of split 1 and 4 (*HES1, HES4*), inhibitor of DNA binding 3 (*ID3*). *Smad 2 *and *Smad3 *are the transcription factors downstream of myostatin/TGF-beta and are able to induce atrophy programming [43,44]. Activation of Notch signaling was able to inhibit myogenesis via transcriptional repressors of *HEY *and *HES *family members [45]. This finding suggests that, in response to RE, along with activation of hypertrophy signaling events, a concomitant activation of atrophic factors may work against a muscle hypertrophic effect in the female muscle.

Furthermore, although muscle hypertrophy was found to be a significantly up-regulated biological theme in both males (4 h and 24 h post exercise) and females (4 h post exercise only), it is noteworthy that sex differences might exist in the fine regulatory system of gene transcription leading to muscle hypertrophy. Specifically, the expression patterns of the mammalian target of rapamycin (mTOR) signaling-associated factors identified in male muscle (24 h post) indicate that there was a coordinated repression of negative regulators of mTOR signaling, which were not observed in females. The mTOR signaling pathway plays a crucial role in mediating muscle hypertrophy [46]. The regulatory mechanisms controlling the activation and inactivation of mTOR signaling in muscle contractile activity are just starting to be elucidated. Several negative regulators have been identified including DNA-damage-inducible transcript 4 (*DDIT4*, also known as *REDD1*), DNA-damage-inducible transcript 4-like (*DDIT4L*, also known as *REDD2*), AKT1 substrate 1 (proline-rich) (*AKT1S1*, also known as *PRAS40*), and tuberous sclerosis 1 and 2 (*TSC1/TSC2*) [47]. In a previous study, Drummond et al. [48] reported decreased mRNA expression of *DDIT4*, *DDIT4L*, *TSC1*, and *TSC2 *in skeletal muscle of young and/or old men after an acute stimulation of protein synthesis via RE and essential amino acid ingestion. Consistent with their reports, our microarray data indicated a significant down-regulation of mTOR inhibitors in male muscle: *DDIT4*, *DDIT4L*, *AKT1S1 *were significantly down-regulated (p < 0.005) and *TSC1 *showed a trend towards a down-regulation in male muscle (24 h post, p = 0.02). However this coordinated negative regulation was not observed in female muscle (only *DDIT4 *showed significant down-regulation) (Figure 3). This finding may implicate a mechanism behind disproportional muscle growth in males as compared with females working at the same relative intensity. Further research using larger sample sizes and measurements of protein content along this pathway is needed to confirm these preliminary findings. Sex differences in these specific features have not been reported elsewhere. Further research is needed to clarify their contribution to sexual dimorphism in muscle phenotypes.

### Limitations of the study

Undoubtedly the sample size in the present study was limited. However, given the fact that human tissue is notoriously hard to obtain in exercise (training) studies, major efforts were utilized to maximize the power of the study by minimizing the potential for experimental error. The technical error inherent in high-density oligonucleotide technology such as the Affymetrix platform is fairly low [27,49]. However, the biological variability is large and has been identified as a major source of experimental error leading to a need for large sample sizes in conducting microarray studies [50]. This is especially true in studies involving human tissue because there is substantial polymorphic noise due to the outbred nature of the human population [27]. Nevertheless, it has been demonstrated that reducing biological variability is an efficient and viable solution for achieving reliable results with a limited number of samples [51]. In the present study, much effort has been paid to reduce the potential influence of inter-individual variability on gene expression, as mentioned at the beginning of the Discussion section.

Furthermore, the analytic methods we used, i.e. LRpath combined with an intensity-based Bayesian moderated t-statistic (IBMT, see the Methods section for details), can maximize the detection power while accurately estimating the false discovery rate, and have also demonstrated superior performance with small sample sizes [21,52]. Consequently, by using a stringent study design and powerful analytical tools, we estimate that we were able to detect a 1.5-fold change at the 0.01 alpha level for 90% of the probes with at least 91% power. For the mid to high expressed genes, the power was even greater due to their lower variance. As such, we believe that the findings from the present study are valid and reliable for three principal reasons: 1) Our results are generally consistent with those reported previously; 2) We studied different individuals at two time points and the major RE-altered biological processes and molecular pathways independently identified were similar across both time points in males; 3) Although we focused on the sex-based differences in gene regulation in this study, it is noteworthy that many of the main biological themes reflected by female and male muscles were similar as expected. The consistent findings within the present study provide additional evidence that our results are reliable.

We also recognize that the changes in protein levels do not always follow transcriptional regulations. Post-transcriptional modification and protein interactions need to be considered in order to fully understand the contribution of these transcriptional findings in future studies.

## Conclusions

In conclusion, our novel data indicate that sex plays an important role in the time course of skeletal muscle transcriptional regulation in response to resistance exercise. The findings from the present study provide insight into our understanding of the mechanisms underlying sex differences in muscle morphology and plasticity. In addition, the plethora of information generated regarding the transcriptionally regulated biological processes aid in understanding the mechanisms underpinning the adaptation of muscle to RE. Ultimately, following final clarification and additional clinical confirmation, these data will provide a knowledge base for refining the exercise prescription guidelines implemented by clinical and fitness professionals in order to optimize targeted fitness and therapeutic goals associated with exercise intervention.

## Methods

### Study overview

Sixteen healthy young men (n = 8) and women (n = 8) were recruited from a large multi-center project, the Functional SNPs Associated with Human Muscle Size and Strength (FAMuSS) study. Expanded details on the FAMuSS study have been published elsewhere [7,17]. The inclusion criteria for participation includes: 1) Between the ages of 18 and 40 y, 2) No chronic diseases, 3) No prior resistance training history, 4) No medications or dietary supplements which may affect musculature. Before joining the present study, all subjects had undergone 24 sessions (2/wk × 12 wks) of supervised progressive unilateral arm resistance exercise training in FAMuSS. The study was conducted in the week following the completion of the FAMuSS training program. In the present study, volunteers repeated their last exercise session of the FAMuSS training. Biceps muscle biopsies were obtained from both arms following the RE session, i.e. the resting untrained and the exercised trained arms from all subjects. Half of the subjects for each sex were randomly selected for muscle biopsy sampling at either 4 h post-exercise, or 24 h post-exercise. Therefore we had equal number of subjects for each sex, representing both early and late recovery responses. Unfortunately, due to unsatisfactory microarray quality, two male subjects, each from 4 h and 24 h, were not included in the microarray analysis. However, both were included in phenotype analysis and follow-up qRT-PCR validation study. The study protocol was approved by the human subjects committee and all the subjects gave their informed consent before joining the study.

### FAMuSS resistance exercise protocol and measurement of muscle mass and strength

The resistance exercise training protocol and measurement of muscle mass and strength implemented by FAMuSS have been reported previously [7,17]. Briefly, each training session consisted of five upper arm exercises (3 sets of 6 RM) with a focus on the elbow flexors including the biceps preacher curl, biceps concentration curl, standing biceps curl, overhead triceps extension, and triceps kickback. Two warm-up sets preceded both the first biceps and triceps exercises. A resting interval of 3 min between warm-up set and testing set and 2 min between testing sets was applied. Isometric strength of the elbow flexor muscles of each arm was determined using a specially constructed, modified preacher bench and strain gauge (model 32628CTL, Lafayette Instrument Company, Lafayette, IN). One repetition maximum (1RM) was measured in a standard preacher curl exercise. Measurement of muscle cross-sectional area was obtained using Magnetic Resonance Imaging (MRI) 48-96 h after the final training session of FAMuSS study to avoid temporary exercise effects. Details about each of the tests can be found in a previously published paper [17].

### Muscle biopsy

Muscle biopsies were obtained from the biceps brachii of both the exercised and resting arms using a percutaneous needle biopsy technique. Briefly, approximately 3-5 cc of lidocaine hydrochloride was used to desensitize the incision area. A ¼ inch diameter University College Hospital (UCH) biopsy needle, with accompanying suction was used to harvest the tissue. Up to 3 passes were allowed in order to ascertain a total of 100 mg of muscle tissue. All biopsy samples were immediately weighed and snap frozen in liquid nitrogen-cooled isopentane, and stored at -80C for subsequent analyses.

### RNA purification and Microarray hybridization

Total RNA was extracted from frozen tissue with a polytron homogenizer (Brinkman, Westbury, NY) and Trizol reagent (Invitrogen, Rockville, MD) and purified with an RNAse kit (Qiagen, Santa Clara, CA). The integrity of total RNA was assessed by running the RNA sample on a denaturing agarose gel stained with ethidium bromide. High quality of RNA was indicated by approximately 2:1 ratio of 28 s and 18 s rRNA bands on the gel. Total RNA was used as a template for double-stranded cDNA synthesis (Superscript Double-Stranded cDNA synthesis kit, Invitrogen, Carlsbad, CA). Biotin-labelled cRNA was synthesized (Enzo Bioarray High Yield RNA transcription Labeling Kit, Affymetrix), and hybridized to Affymetrix (Santa Clara, CA) Affymetrix Human Genome U133 Plus 2.0 arrays according to the manufacturer's instructions. Following hybridization, the probe arrays were washed and stained. The intensity of bound dye was measured with an argon laser confocal scanner (GeneArray Scanner, Agilent). The probe arrays were scanned twice and the stored images were analyzed using the GeneChip software Microarray Analysis Suite (MAS) 5.0 (Affymetrix, Santa Clara, CA). Overall 54675 probe sets representing 20080 annotated genes were profiled.

### Microarray Data Expression and Analysis

The Affymetrix data acquisition programs in MAS 5.0 automatically generate a cell intensity file (CEL) from the stored images that contain a single intensity value for each probe cell on the array. To examine the quality of the various arrays, the R package affyQCreport for generating QC reports was run starting from the CEL files. With the exception of two, all arrays created plots, including the information on percentage of present calls, noise, background, and ratio of glyceraldehydes-3-phosphate dehydrogenase 3' to 5', indicating high quality and an overall comparability of samples. The subjects represented by those two arrays that failed the quality check were removed from the gene expression profile analysis. Raw intensity values of all samples were preprocessed and normalized by RMA using Bioconductor. Differentially expressed genes were tested by using an intensity-based Bayesian moderated t-statistic, IBMT [52]. IBMT is similar to other methods based on hierarchical Bayesian models in testing differential expression of genes in microarray studies. These methods resolve the problem of poor estimation of variance of gene expression due to the small sample size in most microarray studies. Rather than considering each gene/probe separately, these Bayesian modified approaches pool information across genes to achieve a more accurate and stable variance estimation thus improving the results of the tests. IBMT gained further strength as compared with other previously developed methods by incorporating the well-documented information about the dependence of the variance of genes on expression intensity levels. The improved performance of IBMT has been demonstrated in a previously published paper [52], by utilizing simulated data, 'spike-in' Affymetric datasets, and experimental data.

The data discussed in this publication have been deposited in NCBIs Gene Expression Omnibus (GEO, http://www.ncbi.nlm.nih.gov/geo/), and are accessible through GEO Series accession number GSE24235.

### Functional enrichment testing

Enriched Gene Ontology (GO) terms [23] and Kyoto Encyclopaedia of Genes and Genomes (KEGG) pathways [24] were tested by a logistic regression-based method (LRpath), that allows the use of a paired statistical test and has been shown to perform well for experiments with small sample sizes [21]. Enriched GO/KEGG was defined as having a false discovery rate (FDR) less than 0.01. Five comparisons were analyzed including 1) male vs. female biceps in resting state; 2) exercised vs. resting muscle for men at 4 h post; 3) exercised vs. resting muscle for men at 24 h post; 4) exercised vs. resting muscle for women at 4 h post; and 5) exercised vs. resting muscle for women at 24 h post exercise. For this investigation, we employed directional LRpath, which, similar to the Gene Set Enrichment Analysis (GSEA)[20], has the ability to distinguish between up and down regulated gene groups. Directional LRpath tests up- and down- regulated genes simultaneously, and calculates -log(p-value) if the fold change is up, and +log(p-value) if the fold change is down.

Directly related GO terms with considerable overlap of genes were identified for each condition. This is expected because Gene Ontology is structured in such a way that a gene annotated to a child term (more specific term) is also annotated to all its parent terms (less specialized term). In order to alleviate data redundancy, we carefully selected a subset of terms to include in this report by checking the parental relationship between the relevant GO terms. The redundant terms were collapsed by implementing the following strategy: If only the child and parent term were enriched for a similar group of genes, the child term was used; if sibling terms and parent term were all involved, the more generalized parent term was used. In addition, although three sub-ontologies of GO, i.e. cellular component, biological process and molecular function, were considered and tested, our emphasis was given to biological processes because, along with KEGG pathway, biological processes were more relevant to the purpose of our study to reveal cellular events responsible for skeletal muscle response and adaptation to exercise.

### Real-time RT-PCR

Quantitative reverse transcriptase-polymerase chain reaction (qRT-PCR) was used to validate a subset of genes expressed differentially based on the microarray results. One or two genes were selected from interesting functional categories revealed by the microarray analysis as significantly differentially regulated by RE. Following completion of the microarray experiment, muscle biopsy samples were only available for a subset of subjects (n = 2 for each of the four groups: male/female, 4 h and 24 h post exercise). Total RNA was isolated from these samples with TRIzol reagent (Invitrogen, Carlsbad, CA). The quality and quantity of the total RNA samples was checked using a spectrophotometer (NanoDrop, ND-1000, Thermo Fisher Scientific, Inc., Wilmington, DE) prior to reverse transcription. The OD 260/280 ratio of our samples ranged from 1.84~1.97 indicating good quality. Random hexamers were used to generate single-strand cDNA using MMLV Reverse Transcriptase according to the manufacturer's protocol (Invitrogen). cDNA was cleaned using QIAquick PCR purification kit (Qiagen, Carlsbad, CA). The qRT-PCR was performed using an Applied Biosystems Taqman Gene Expression Assay containing a FAM dye-labeled Taqman MGB probe on the Applied Biosystems 7500 Real-Time PCR System (Applied Biosystems, Life Technologies Corporation, Carlsbad, CA). The selected genes and TaqMan assays are displayed in supplementary data (Additional file 9, Table S8). The reaction was prepared according to the standard Taqman gene expression assay protocol in a total volume of 20 μl (Applied Biosystems). Thermo cycling conditions were standard and as follows: 10 min at 95°C followed by 40 cycles of 15 s at 95°C and 1 min at 60°C for annealing. All samples were analyzed in duplicate. Eukaryotic beta 2-microglobulin (*B2M*) was included as an internal control to calibrate the expression levels of target gene [53]. The validity of *B2 M *as an internal control in acute resistance exercise studies in human skeletal muscle has been previously tested and confirmed[53]. After amplification, all data were normalized to *B2M*, and the relative changes in gene expression between exercised and control muscle was calculated using the 2^ΔΔCt method [54]. As a prerequisite assumption of using the 2^ΔΔCt method for analyses, the consistency of the amplification efficiency of *B2 M *and all the target genes were tested via serial dilution and confirmed in two randomly selected samples.

To validate the microarray data, correlation was tested for fold changes measured by microarray and qPCR for all the selected genes. For the genes represented with multiple probes in microarray, the probe with the highest average signal intensity was used. Since the data was not normally distributed (Shapiro-Wilk test, p < 0.05), Spearman's Rho was used. For the microarray, the data input into the correlation analysis was the weighted average of the fold change for each gene on the array representing all replicate individuals. For qRT-PCR, we used the mean fold change calculated as 2^ΔΔCt for all replicate individuals.

### Statistical analysis

Data on physical characteristics of subjects are reported as mean ± SE or median (interquartile range), sex differences were tested either by t-test, or by Wilcoxon Rank Sum Test when the assumption of normality was violated, and statistical significance was set at p < 0.05. Differentially expressed genes were tested using IBMT. Enrichment analysis for GO terms and KEGG pathways was conducted by using LRpath. Multiple testing correction was done by using False Discovery Rates (FDR) approach [55], and significantly enriched concepts (GO or KEGG) were defined as having FDR < 0.01. FDR approach, as compared with the classic use of family-wide error rate (FWER), is less stringent, but offers substantial gain in power especially when a large number of the non-true null hypotheses are expected [56]. The qRT- PCR data were analyzed using Spearman's Rho test for correlation with microarray data. Analysis of covariance was used to compare mRNA levels (Ct values) of male and female control muscles using Ct value of B2 M as a covariate. p < 0.05 was accepted as significant in both tests. IBMT and LRpath were performed using R. Other analyses were conducted using SAS 9.1 (SAS Institute, Cary, NC).

## Authors' contributions

The project was conceived and supervised by PMG. Data analysis was carried out by MS and DL. The qRT-PCR was done by MKT and HBI. The microarray gene expression experiments were done by GN. DL and PMG wrote the manuscript with input from all authors. All authors read and approved the final manuscript.

## Supplementary Material

Additional file 1 Table S1: Gene concepts enriched with sex-related differential expression genes. Click here for file

Additional file 2 Table S2: Significantly enriched biological concepts for up- and down-regulated genes in male biceps 4 h post-RE. Click here for file

Additional file 3 Table S3: Enriched biological concepts for up- and down-regulated genes in male biceps 24 h post-RE. Click here for file

Additional file 4 Table S4: Enriched biological concepts for up- and down-regulated genes in female biceps 4 h post-RE. Click here for file

Additional file 5 Table S5: Enriched biological concepts for up- and down-regulated genes in female biceps 24 h post-RE. Click here for file

Additional file 6 Table S6: Differential expression of the selected genes in exercised vs. rested muscle measured by qRT-PCR. Click here for file

Additional file 7 Table S7: Key genes involved in RE-induced transcriptional regulation in male and female biceps branchii. Click here for file

Additional file 8 **Discussion on common GO terms and KEGGs pathways regulated by RE in both male and female biceps**. Click here for file

Additional file 9 Table S8: Genes for qRT-PCR validation and TaqMan assay used. Click here for file
