# Supplementary material for: Skeletal muscle gene expression in response to resistance exercise: sex specific regulation
Source: BMC Genomics. 2010 Nov 24;11:659. doi: 10.1186/1471-2164-11-659 (PMC3091777; doi:10.1186/1471-2164-11-659)
Supplement: Additional file 1 — Table S1: Gene concepts enriched with sex-related differential expression genes. [file 1471-2164-11-659-S1.DOCX]

| **Table S1. Gene concepts enriched with sex-related differential expression genes.** | | | | | |
| --- | --- | --- | --- | --- | --- |
| KEGG pathways and GO terms having *FDR<0.01* from LRpath analysis are shown (redundant GO terms were collapsed based on substantial overlap of genes and/or parent-child relationship between relevant GO terms). *Odds ratios* were calculated based on the difference between a *p-value*=0.50 and a *p-value*=0.001; *gene #* indicates how many analyzed genes belong to each enriched category; *p Value* indicates significance of enrichment testing by LRpath analysis; *FDR*, False Discovery Rates, significance statistic adjusted for multiple testing. | | | | | |
|  | | | | | |
| Concept ID | Concept Name | Gene # | Odds Ratio | p Value | FDR |
| **Concepts enriched with female-associated high expression genes** | | | | | |
| hsa03018 | RNA degradation | 56 | 0.08 | 6.86E-06 | 1.19E-03 |
| hsa03010 | Ribosome | 84 | 0.12 | 1.26E-05 | 1.19E-03 |
| hsa03022 | Basal transcription factors | 34 | 0.06 | 2.14E-05 | 1.35E-03 |
| hsa00071 | Fatty acid metabolism | 41 | 0.07 | 3.73E-05 | 1.77E-03 |
| hsa00720 | Reductive carboxylate cycle (CO2 fixation) | 10 | 0.02 | 2.22E-04 | 8.45E-03 |
| GO:0006396 | RNA processing | 525 | 0.27 | 2.48E-09 | 7.02E-07 |
| GO:0019395 | Fatty acid oxidation | 45 | 0.07 | 1.52E-06 | 2.67E-04 |
| GO:0000123 | Histone acetyltransferase complex | 50 | 0.11 | 8.16E-05 | 5.67E-03 |
| GO:0006367 | Transcription initiation from RNA polymerase II promoter | 71 | 0.14 | 9.72E-05 | 6.28E-03 |
| **Concepts enriched with male-associated high expression genes** | | | | | |
| GO:0070011 | Peptidase activity, acting on L-amino acid peptides | 506 | 2.09 | 2.49E-05 | 2.24E-03 |
| GO:0007283 | Spermatogenesis | 236 | 2.34 | 7.71E-05 | 5.56E-03 |
| GO:0070646 | Protein modification by small protein removal | 27 | 3.44 | 9.07E-05 | 6.00E-03 |
