# Supplementary material for: Skeletal muscle gene expression in response to resistance exercise: sex specific regulation
Source: BMC Genomics. 2010 Nov 24;11:659. doi: 10.1186/1471-2164-11-659 (PMC3091777; doi:10.1186/1471-2164-11-659)
Supplement: Additional file 2 — Table S2: Significantly enriched biological concepts for up- and down-regulated genes in male biceps 4 h post-RE. [file 1471-2164-11-659-S2.DOCX]

| **Table S2. Significantly enriched biological concepts for up- and down-regulated genes in male biceps 4h post-RE.**  KEGG pathways and GO terms having *FDR<0.01* from LRpath analysis are shown (redundant GO terms were collapsed based on substantial overlap of genes and/or parent-child relationship between relevant GO terms). *Odds ratios* were calculated based on the difference between a *p-value*=0.50 and a *p-value*=0.001; *gene #* indicates how many analyzed genes belong to each enriched category; *p Value* indicates significance of enrichment testing by LRpath analysis; *FDR*, False Discovery Rates, significance statistic adjusted for multiple testing. | | | | | | | | |
| --- | --- | --- | --- | --- | --- | --- | --- | --- |
| Concept ID | Concept Name | Gene # | | Odds Ratio | P Value | | | FDR |
| **Concepts enriched with up-regulated genes** | | | | | | | | |
|  | **ECM and cytoskeleton based processes** | | | |  | | |  |
| hsa04512 | ECM-receptor interaction | 84 | | 0.06 | 2.43E-09 | | | 4.48E-07 |
| hsa04510 | Focal adhesion | 199 | | 0.14 | 4.71E-09 | | | 4.48E-07 |
| GO:0031012 | Extracellular matrix | 316 | | 0.20 | 1.58E-10 | | | 1.63E-08 |
| GO:0007155 | Cell adhesion | 721 | | 0.34 | 8.76E-10 | | | 7.56E-08 |
| GO:0016477 | Cell migration | 297 | | 0.23 | 1.58E-08 | | | 1.06E-06 |
| GO:0007015 | Actin filament organization | 106 | | 0.13 | 1.46E-06 | | | 5.74E-05 |
| GO:0030036 | Actin cytoskeleton organization | 233 | | 0.27 | 1.17E-05 | | | 3.54E-04 |
| GO:0007229 | Integrin-mediated signaling pathway | 55 | | 0.12 | 2.76E-04 | | | 4.77E-03 |
| GO:0030030 | Cell projection organization | 277 | | 0.39 | 6.58E-04 | | | 9.62E-03 |
|  | **Neuromuscular junction** |  | |  |  | | |  |
| hsa04080 | Neuroactive ligand-receptor interaction | 255 | | 0.31 | 1.05E-04 | | | 4.11E-03 |
| GO:0007399 | Nervous system development | 814 | | 0.49 | 1.58E-05 | | | 4.49E-04 |
|  | **Muscle hypertrophy** |  | |  |  | | |  |
| GO:0009888 | Tissue development | 546 | | 0.29 | 5.01E-10 | | | 4.86E-08 |
| GO:0008284 | Positive regulation of cell proliferation | 309 | | 0.33 | 2.21E-05 | | | 5.89E-04 |
| GO:0000904 | Cell morphogenesis involved in differentiation | 195 | | 0.28 | 1.15E-04 | | | 2.39E-03 |
| GO:0060537 | Muscle tissue development | 127 | | 0.21 | 1.03E-04 | | | 2.16E-03 |
| GO:0032989 | Cellular component morphogenesis | 323 | | 0.40 | 3.21E-04 | | | 5.42E-03 |
|  | **Angiogenesis** |  | |  |  | | |  |
| GO:0001944 | Vasculature development | 229 | | 0.19 | 3.33E-08 | | | 2.15E-06 |
|  | **Signal transduction** |  | |  |  | | |  |
| GO:0017017 | MAP kinase tyrosine/serine/threonine phosphatase activity | 13 | | 0.01 | 1.10E-07 | | | 6.30E-06 |
| GO:0007186 | G-protein coupled receptor protein signaling pathway | 698 | | 0.46 | 1.41E-05 | | | 4.14E-04 |
| GO:0007264 | Small GTPase mediated signal transduction | 470 | | 0.44 | 1.47E-04 | | | 2.91E-03 |
| GO:0007167 | Enzyme linked receptor protein signaling pathway | 374 | | 0.43 | 3.51E-04 | | | 5.72E-03 |
| GO:0004859 | Phospholipase inhibitor activity | 12 | | 0.01 | 1.43E-05 | | | 4.17E-04 |
| GO:0009966 | Regulation of signal transduction | 700 | | 0.49 | 6.59E-05 | | | 1.46E-03 |
| GO:0019935 | Cyclic-nucleotide-mediated signaling | 106 | | 0.21 | 2.70E-04 | | | 4.68E-03 |
| GO:0046058 | cAMP metabolic process | 92 | | 0.20 | 6.24E-04 | | | 9.26E-03 |
| GO:0019932 | Second-messenger-mediated signaling | 209 | | 0.23 | 1.89E-06 | | | 7.11E-05 |
|  | **Ion transport** |  | |  |  | | |  |
| GO:0001503 | Ossification | 125 | | 0.17 | 6.92E-06 | | | 2.25E-04 |
| GO:0005509 | Calcium ion binding | 866 | | 0.49 | 1.09E-05 | | | 3.31E-04 |
| GO:0006811 | Ion transport | 739 | | 0.51 | 1.17E-04 | | | 2.43E-03 |
| GO:0031420 | Alkali metal ion binding | 220 | | 0.33 | 3.43E-04 | | | 5.69E-03 |
|  | **Response to stimuli and inflammation** | | | | | | |  |
| GO:0050921 | Positive regulation of chemotaxis | 22 | | 0.03 | 1.81E-05 | | | 5.01E-04 |
|  | **Transcrition and translation** |  | |  |  | | |  |
| GO:0003700 | Transcription factor activity | 893 | | 0.52 | 4.25E-05 | | | 1.03E-03 |
| **Concepts enriched with down-regulated genes** | | | | | | | | |
|  | **Gene transcription and Translation** |  |  | | |  |  | |
| hsa00970 | Aminoacyl-tRNA biosynthesis | 41 | 9.59 | | | 1.74E-04 | 5.52E-03 | |
| GO:0005840 | Ribosome | 187 | 8.92 | | | 1.32E-13 | 2.56E-11 | |
| GO:0004540 | Ribonuclease activity | 55 | 10.49 | | | 8.02E-06 | 2.59E-04 | |
| GO:0006412 | Translation | 379 | 4.36 | | | 3.80E-11 | 4.23E-09 | |
| GO:0045182 | Translation regulator activity | 110 | 4.17 | | | 4.58E-04 | 7.23E-03 | |
| GO:0006368 | RNA elongation from RNA polymerase II promoter | 46 | 7.88 | | | 4.88E-04 | 7.58E-03 | |
| GO:0006325 | Chromatin organization | 353 | 2.49 | | | 1.28E-04 | 2.60E-03 | |
| GO:0006396 | RNA processing | 525 | 3.45 | | | 1.59E-10 | 1.63E-08 | |
| GO:0005681 | Spliceosomal complex | 130 | 7.70 | | | 1.03E-08 | 7.32E-07 | |
| GO:0022613 | Ribonucleoprotein complex biogenesis | 170 | 3.89 | | | 3.98E-05 | 9.77E-04 | |
| GO:0008168 | Methyltransferase activity | 163 | 4.92 | | | 1.52E-06 | 5.89E-05 | |
| GO:0008156 | Negative regulation of DNA replication | 25 | 14.67 | | | 3.32E-04 | 5.54E-03 | |
|  | **Protein metabolic process** |  |  | | |  |  | |
| hsa00280 | Valine, leucine and isoleucine degradation | 43 | 8.83 | | | 2.43E-04 | 6.59E-03 | |
| hsa00350 | Tyrosine metabolism | 46 | 7.87 | | | 3.90E-04 | 8.23E-03 | |
| GO:0006520 | Cellular amino acid metabolic process | 221 | 2.76 | | | 6.51E-04 | 9.55E-03 | |
| GO:0006457 | Protein folding | 161 | 4.29 | | | 1.57E-05 | 4.49E-04 | |
| GO:0051186 | Cofactor metabolic process | 172 | 4.72 | | | 1.67E-06 | 6.33E-05 | |
|  | **Mitochondrial part and oxidative phosphorylation** | | | | |  |  | |
| GO:0044429 | Mitochondrial part | 528 | 4.51 | | | 2.55E-15 | 9.39E-13 | |
| GO:0005761 | Mitochondrial ribosome | 46 | 36.40 | | | 9.67E-13 | 1.27E-10 | |
| GO:0016491 | Oxidoreductase activity | 644 | 2.80 | | | 6.74E-09 | 4.96E-07 | |
| GO:0042180 | Cellular ketone metabolic process | 527 | 2.06 | | | 2.63E-04 | 4.63E-03 | |
| GO:0045730 | Respiratory burst | 15 | 60.91 | | | 5.52E-07 | 2.51E-05 | |
|  | **Stress response** |  |  | | |  |  | |
| GO:0006968 | Cellular defense response | 58 | 8.52 | | | 4.31E-05 | 1.04E-03 | |
| GO:0032760 | Positive regulation of tumor necrosis factor production | 10 | 54.23 | | | 8.06E-05 | 1.75E-03 | |
