# Supplementary material for: Skeletal muscle gene expression in response to resistance exercise: sex specific regulation
Source: BMC Genomics. 2010 Nov 24;11:659. doi: 10.1186/1471-2164-11-659 (PMC3091777; doi:10.1186/1471-2164-11-659)
Supplement: Additional file 3 — Table S3: Enriched biological concepts for up- and down-regulated genes in male biceps 24 h post-RE. [file 1471-2164-11-659-S3.DOCX]

| **Table S3. Enriched biological concepts for up- and down-regulated genes in male biceps 24h post-RE.** | | | | | | | |
| --- | --- | --- | --- | --- | --- | --- | --- |
| KEGG pathways and GO terms having *FDR<0.01* from LRpath analysis are shown (redundant GO terms were collapsed based on substantial overlap of genes and/or parent-child relationship between relevant GO terms). *Odds ratios* were calculated based on the difference between a *p-value*=0.50 and a *p-value*=0.001; *gene #* indicates how many analyzed genes belong to each enriched category; *p Value* indicates significance of enrichment testing by LRpath analysis; *FDR*, False Discovery Rates, significance statistic adjusted for multiple testing. | | | | | | | |
|  | | | | | | | |
| Concept ID | Concept Name | Gene # | | Odds Ratio | p Value | FDR | |
| **Concepts enriched with up-regulated genes** | | | | | | | |
|  | **ECM and cytoskeleton based process** |  | |  |  |  | |
| hsa04512 | ECM-receptor interaction | 84 | | 0.21 | 1.23E-15 | 2.34E-13 | |
| hsa04540 | Gap junction | 89 | | 0.35 | 9.60E-07 | 3.65E-05 | |
| hsa04510 | Focal adhesion | 199 | | 0.34 | 4.39E-12 | 2.78E-10 | |
| hsa04810 | Regulation of actin cytoskeleton | 211 | | 0.54 | 2.37E-04 | 4.09E-03 | |
| GO:0030198 | Extracellular matrix organization | 74 | | 0.25 | 3.68E-15 | 5.41E-13 | |
| GO:0005578 | Proteinaceous extracellular matrix | 298 | | 0.35 | 1.04E-19 | 6.38E-17 | |
| GO:0032964 | Collagen biosynthetic process | 12 | | 0.18 | 4.01E-08 | 2.14E-06 | |
| GO:0009897 | External side of plasma membrane | 91 | | 0.46 | 4.44E-04 | 7.43E-03 | |
| GO:0007015 | Actin filament organization | 106 | | 0.47 | 2.28E-04 | 4.38E-03 | |
| GO:0042641 | Actomyosin | 20 | | 0.3 | 3.70E-04 | 6.39E-03 | |
| GO:0001502 | Cartilage condensation | 12 | | 0.22 | 2.14E-05 | 6.28E-04 | |
| GO:0007155 | Cell adhesion | 721 | | 0.54 | 3.54E-12 | 3.34E-10 | |
| GO:0005921 | Gap junction | 24 | | 0.31 | 2.56E-04 | 4.82E-03 | |
| GO:0005539 | Glycosaminoglycan binding | 123 | | 0.48 | 1.83E-04 | 3.60E-03 | |
| GO:0030203 | Glycosaminoglycan metabolic process | 58 | | 0.39 | 2.01E-04 | 3.93E-03 | |
| GO:0018298 | Protein-chromophore linkage | 14 | | 0.26 | 2.83E-04 | 5.15E-03 | |
|  | **Cell death** |  | |  |  |  | |
| GO:0006915 | Apoptosis | 879 | | 0.71 | 6.21E-05 | 1.44E-03 | |
|  | **Angiogenesis** |  | |  |  |  | |
| GO:0001568 | Blood vessel development | 225 | | 0.46 | 5.72E-08 | 3.01E-06 | |
|  | **Signal transduction** |  | |  |  |  | |
| hsa04115 | p53 signaling pathway | 68 | | 0.39 | 2.11E-04 | 4.02E-03 | |
| GO:0007229 | Integrin-mediated signaling pathway | 55 | | 0.37 | 5.79E-05 | 1.36E-03 | |
| GO:0005509 | Calcium ion binding | 866 | | 0.69 | 3.11E-05 | 8.30E-04 | |
| GO:0006182 | cGMP biosynthetic process | 15 | | 0.27 | 4.59E-04 | 7.60E-03 | |
| GO:0005525 | GTP binding | 347 | | 0.52 | 6.34E-08 | 3.29E-06 | |
| GO:0005834 | Heterotrimeric G-protein complex | 26 | | 0.32 | 4.73E-04 | 7.81E-03 | |
| GO:0004859 | Phospholipase inhibitor activity | 12 | | 0.22 | 1.01E-05 | 3.24E-04 | |
| GO:0016023 | Cytoplasmic membrane-bounded vesicle | 410 | | 0.65 | 4.15E-04 | 7.00E-03 | |
| GO:0008329 | Pattern recognition receptor activity | 14 | | 0.27 | 5.34E-04 | 8.50E-03 | |
|  | **Tissue development and remodeling** |  | |  |  |  | |
| hsa05222 | Small cell lung cancer | 86 | | 0.44 | 5.06E-04 | 8.01E-03 | |
| GO:0043623 | Cellular protein complex assembly | 191 | | 0.52 | 5.71E-05 | 1.36E-03 | |
| GO:0051128 | Regulation of cellular component organization | 367 | | 0.6 | 5.78E-05 | 1.36E-03 | |
| GO:0008284 | Positive regulation of cell proliferation | 309 | | 0.58 | 5.51E-05 | 1.33E-03 | |
| GO:0022604 | Regulation of cell morphogenesis | 91 | | 0.44 | 1.36E-04 | 2.92E-03 | |
| GO:0043588 | Skin development | 18 | | 0.22 | 3.65E-07 | 1.60E-05 | |
| GO:0016044 | Membrane organization | 358 | | 0.61 | 1.35E-04 | 2.91E-03 | |
|  | **Growth factor activity** |  | |  |  |  | |
| GO:0019838 | Growth factor binding | 94 | | 0.46 | 2.78E-04 | 5.15E-03 | |
| GO:0005520 | Insulin-like growth factor binding | 25 | | 0.32 | 3.93E-04 | 6.69E-03 | |
|  | **Stress response and immune activation** |  | |  |  |  | |
| hsa04670 | Leukocyte transendothelial migration | 115 | | 0.44 | 8.79E-05 | 2.39E-03 | |
| hsa05130 | Pathogenic Escherichia coli infection - EHEC | 49 | | 0.17 | 5.44E-15 | 5.17E-13 | |
| GO:0006955 | Immune response | 654 | | 0.65 | 1.43E-05 | 4.34E-04 | |
| GO:0006928 | Cell motion | 472 | | 0.54 | 8.13E-09 | 4.68E-07 | |
| GO:0016477 | Cell migration | 297 | | 0.49 | 3.61E-08 | 1.96E-06 | |
| GO:0030336 | Negative regulation of cell migration | 50 | | 0.39 | 4.99E-04 | 8.19E-03 | |
| GO:0050900 | Leukocyte migration | 57 | | 0.4 | 3.62E-04 | 6.28E-03 | |
| GO:0008191 | Metalloendopeptidase inhibitor activity | 10 | | 0.21 | 2.80E-05 | 7.51E-04 | |
| GO:0042470 | Melanosome | 86 | | 0.42 | 4.60E-05 | 1.15E-03 | |
| GO:0009607 | Response to biotic stimulus | 313 | | 0.6 | 1.41E-04 | 2.99E-03 | |
| GO:0006986 | Response to unfolded protein (heat shock protein activity ) | 67 | | 0.35 | 9.77E-07 | 3.79E-05 | |
| GO:0030968 | Endoplasmic reticulum unfolded protein response | 19 | | 0.26 | 2.15E-05 | 6.28E-04 | |
| GO:0009611 | Response to wounding | 468 | | 0.67 | 5.33E-04 | 8.50E-03 | |
|  | **Muscle hypertrophy** |  | |  |  |  | |
| GO:0045445 | Myoblast differentiation | 23 | | 0.31 | 4.40E-04 | 7.39E-03 | |
| GO:0070271 | Protein complex biogenesis | 381 | | 0.55 | 8.38E-07 | 3.28E-05 | |
| GO:0005198 | Structural molecule activity | 572 | | 0.63 | 7.80E-06 | 2.56E-04 | |
| GO:0009119 | Ribonucleoside metabolic process | 41 | | 0.35 | 1.48E-04 | 3.08E-03 | |
| GO:0044259 | Multicellular organismal macromolecule metabolic process | 37 | | 0.33 | 4.64E-05 | 1.15E-03 | |
| **Concepts enriched with down-regulated genes** | | | | | | | |
|  | **Fatty acid metabolism** | |  | |  | |  |
| hsa00071 | Fatty acid metabolism | 41 | 3.77 | | 8.22E-06 | | 2.60E-04 |
| GO:0046463 | Acylglycerol biosynthetic process | 13 | 6.26 | | 2.36E-05 | | 6.80E-04 |
| GO:0019395 | Fatty acid oxidation | 45 | 4.06 | | 1.76E-06 | | 6.42E-05 |
| GO:0045923 | Positive regulation of fatty acid metabolic process | 15 | 5.83 | | 2.64E-05 | | 7.35E-04 |
|  | **Amino acid metabolism** | |  | |  | |  |
| GO:0009081 | Branched chain family amino acid metabolic process | 16 | 5.46 | | 5.25E-05 | | 1.27E-03 |
| GO:0019941 | Modification-dependent protein catabolic process | 550 | 2.01 | | 3.38E-10 | | 2.35E-08 |
| hsa00280 | Valine, leucine and isoleucine degradation | 43 | 4.31 | | 1.94E-07 | | 9.23E-06 |
|  | **Carbohydrate metabolism** | |  | |  | |  |
| hsa00650 | Butanoate metabolism | 33 | 3.62 | | 1.05E-04 | | 2.50E-03 |
| hsa00640 | Propanoate metabolism | 32 | 3.63 | | 1.19E-04 | | 2.51E-03 |
| GO:0005977 | Glycogen metabolic process | 44 | 3.39 | | 1.29E-04 | | 2.82E-03 |
|  | **Transcription and translation** | |  | |  | |  |
| GO:0016568 | Chromatin modification | 268 | 2.15 | | 6.06E-07 | | 2.53E-05 |
| GO:0051276 | Chromosome organization | 448 | 1.68 | | 3.22E-05 | | 8.36E-04 |
| GO:0044451 | Nucleoplasm part | 455 | 1.85 | | 6.30E-07 | | 2.61E-05 |
| GO:0045941 | Positive regulation of transcription | 437 | 1.55 | | 6.21E-04 | | 9.68E-03 |
| GO:0006471 | Protein amino acid ADP-ribosylation | 22 | 4.49 | | 1.44E-04 | | 3.03E-03 |
| GO:0006366 | Transcription from RNA polymerase II promoter | 748 | 1.5 | | 5.22E-05 | | 1.27E-03 |
|  | **Mitochondrial part and oxidative phosphorylation** | |  | |  | |  |
| GO:0015980 | Energy derivation by oxidation of organic compounds | 139 | 2.08 | | 5.62E-04 | | 8.92E-03 |
| GO:0044429 | Mitochondrial part | 528 | 1.58 | | 1.00E-04 | | 2.25E-03 |
| GO:0055114 | Oxidation reduction | 579 | 1.64 | | 9.94E-06 | | 3.24E-04 |
| GO:0016491 | Oxidoreductase activity | 644 | 1.48 | | 2.72E-04 | | 5.08E-03 |
|  | **Muscle contraction** | |  | |  | |  |
| GO:0003009 | Skeletal muscle contraction | 10 | 5.78 | | 5.92E-04 | | 9.35E-03 |
| GO:0016529 | Sarcoplasmic reticulum | 32 | 3.55 | | 5.28E-04 | | 8.49E-03 |
| GO:0042809 | Vitamin D receptor binding | 21 | 4.4 | | 2.81E-04 | | 5.15E-03 |
