# Supplementary material for: Skeletal muscle gene expression in response to resistance exercise: sex specific regulation
Source: BMC Genomics. 2010 Nov 24;11:659. doi: 10.1186/1471-2164-11-659 (PMC3091777; doi:10.1186/1471-2164-11-659)
Supplement: Additional file 4 — Table S4: Enriched biological concepts for up- and down-regulated genes in female biceps 4 h post-RE. [file 1471-2164-11-659-S4.DOCX]

| **Table S4. Enriched biological concepts for up- and down-regulated genes in female biceps 4h post-RE.** | | | | | | | | |
| --- | --- | --- | --- | --- | --- | --- | --- | --- |
| KEGG pathways and GO terms having *FDR<0.01* from LRpath analysis are shown (redundant GO terms were collapsed based on substantial overlap of genes and/or parent-child relationship between relevant GO terms). *Odds ratios* were calculated based on the difference between a *p-value*=0.50 and a *p-value*=0.001; *gene #* indicates how many analyzed genes belong to each enriched category; *p Value* indicates significance of enrichment testing by LRpath analysis; *FDR*, False Discovery Rates, significance statistic adjusted for multiple testing. | | | | | | | | |
|  | | | | | | | | |
| Concept ID | Concept Name | Gene # | | | Odds Ratio | p Value | | FDR |
| **Concepts enriched with up-regulated genes** | | | | | | | | |
|  | **ECM and cytoskeleton based processes** | | | |  |  | |  |
| hsa04510 | Focal adhesion | 199 | | | 0.22 | 1.21E-24 | | 2.30E-22 |
| hsa04512 | ECM-receptor interaction | 84 | | | 0.17 | 1.27E-21 | | 1.20E-19 |
| hsa04540 | Gap junction | 89 | | | 0.33 | 1.82E-07 | | 5.77E-06 |
| hsa04520 | Adherens junction | 75 | | | 0.34 | 1.99E-06 | | 3.78E-05 |
| hsa04810 | Regulation of actin cytoskeleton | 211 | | | 0.47 | 3.19E-06 | | 5.50E-05 |
| hsa00532 | Chondroitin sulfate biosynthesis | 22 | | | 0.24 | 3.93E-05 | | 4.39E-04 |
| hsa04530 | Tight junction | 131 | | | 0.47 | 1.13E-04 | | 1.03E-03 |
| hsa04514 | Cell adhesion molecules (CAMs) | 130 | | | 0.49 | 3.93E-04 | | 3.11E-03 |
| GO:0031012 | Extracellular matrix | 316 | | | 0.28 | 7.11E-32 | | 1.31E-28 |
| GO:0007155 | Cell adhesion | 721 | | | 0.41 | 1.72E-24 | | 3.96E-22 |
| GO:0030199 | Collagen fibril organization | 25 | | | 0.12 | 2.22E-17 | | 3.03E-15 |
| GO:0005913 | Cell-cell adherens junction | 20 | | | 0.19 | 1.84E-07 | | 4.31E-06 |
| GO:0005925 | Focal adhesion | 92 | | | 0.44 | 2.74E-04 | | 2.42E-03 |
| GO:0005921 | Gap junction | 24 | | | 0.29 | 5.21E-04 | | 4.20E-03 |
| GO:0070161 | Anchoring junction | 153 | | | 0.42 | 3.79E-07 | | 8.06E-06 |
| GO:0030036 | Actin cytoskeleton organization/ | 233 | | | 0.41 | 3.82E-10 | | 1.55E-08 |
| GO:0003779 | Actin binding | 304 | | | 0.42 | 6.79E-12 | | 3.77E-10 |
| GO:0030832 | Regulation of actin filament length | 55 | | | 0.4 | 8.70E-04 | | 6.52E-03 |
| GO:0008305 | Integrin complex | 28 | | | 0.3 | 5.19E-04 | | 4.19E-03 |
| GO:0001725 | Stress fiber | 17 | | | 0.21 | 1.56E-05 | | 2.15E-04 |
| GO:0005518 | Collagen binding | 31 | | | 0.17 | 3.58E-12 | | 2.13E-10 |
| GO:0005178 | Integrin binding | 51 | | | 0.26 | 3.28E-08 | | 9.35E-07 |
| GO:0005539 | Glycosaminoglycan binding | 123 | | | 0.38 | 1.42E-07 | | 3.51E-06 |
| GO:0009986 | Cell surface | 237 | | | 0.44 | 7.77E-09 | | 2.49E-07 |
| GO:0031252 | Cell leading edge | 109 | | | 0.36 | 1.54E-07 | | 3.71E-06 |
| GO:0050654 | Chondroitin sulfate proteoglycan metabolic process | 14 | | | 0.22 | 1.97E-04 | | 1.85E-03 |
| GO:0018298 | Protein-chromophore linkage | 14 | | | 0.24 | 7.46E-04 | | 5.73E-03 |
|  | **Signal transduction** | | | |  |  | |  |
| hsa04010 | MAPK signaling pathway | | 268 | | 0.46 | 5.96E-08 | | 2.33E-06 |
| hsa04350 | TGF-beta signaling pathway | | 84 | | 0.45 | 7.69E-04 | | 5.07E-03 |
| GO:0009966 | Regulation of signal transduction | | 700 | | 0.57 | 1.87E-09 | | 6.62E-08 |
| GO:0006469 | Negative regulation of protein kinase activity | | 72 | | 0.38 | 4.21E-05 | | 4.90E-04 |
| GO:0007167 | Enzyme linked receptor protein signaling pathway | | 374 | | 0.49 | 3.82E-09 | | 1.31E-07 |
| GO:0007169 | Transmembrane receptor protein tyrosine kinase signaling | | 240 | | 0.49 | 1.05E-06 | | 1.98E-05 |
| GO:0007178 | Transmembrane receptor protein serine/threonine kinase signaling | | 123 | | 0.44 | 2.22E-05 | | 2.86E-04 |
| GO:0010627 | Regulation of protein kinase cascade | | 213 | | 0.54 | 1.27E-04 | | 1.29E-03 |
| GO:0007229 | Integrin-mediated signaling pathway | | 55 | | 0.35 | 6.99E-05 | | 7.71E-04 |
| GO:0017017 | MAP kinase tyrosine/serine/threonine phosphatase activity | | 13 | | 0.19 | 3.73E-05 | | 4.43E-04 |
| GO:0007264 | Small GTPase mediated signal transduction | | 470 | | 0.6 | 6.30E-06 | | 9.58E-05 |
| GO:0003924 | GTPase activity | | 187 | | 0.48 | 9.55E-06 | | 1.40E-04 |
| GO:0019897 | Extrinsic to plasma membrane | | 42 | | 0.33 | 1.66E-04 | | 1.63E-03 |
| GO:0009725 | Response to hormone stimulus | | 189 | | 0.54 | 3.53E-04 | | 3.02E-03 |
| GO:0009190 | Cyclic nucleotide biosynthetic process | | 100 | | 0.48 | 1.05E-03 | | 7.51E-03 |
| GO:0009975 | Cyclase activity | | 22 | | 0.27 | 2.64E-04 | | 2.37E-03 |
| GO:0005160 | Transforming growth factor beta receptor binding | | 17 | | 0.25 | 4.17E-04 | | 3.50E-03 |
| GO:0004866 | Endopeptidase inhibitor activity | | 132 | | 0.49 | 3.17E-04 | | 2.77E-03 |
| GO:0009967 | Positive regulation of signal transduction | | 243 | | 0.58 | 3.56E-04 | | 3.04E-03 |
| GO:0051591 | Response to cAMP | | 11 | | 0.2 | 4.19E-04 | | 3.50E-03 |
| GO:0005509 | Calcium ion binding | | 866 | | 0.58 | 1.35E-10 | | 5.94E-09 |
| GO:0046332 | SMAD binding | | 38 | | 0.33 | 2.71E-04 | | 2.42E-03 |
| GO:0005516 | Calmodulin binding | | 136 | | 0.49 | 2.73E-04 | | 2.42E-03 |
| GO:0007259 | JAK-STAT cascade | | 58 | | 0.35 | 5.70E-05 | | 6.58E-04 |
| GO:0007219 | Notch signaling pathway | | 51 | | 0.34 | 6.98E-05 | | 7.71E-04 |
| GO:0017124 | SH3 domain binding | | 91 | | 0.47 | 1.24E-03 | | 8.68E-03 |
| GO:0010524 | Positive regulation of calcium ion transport into cytosol | | 12 | | 0.22 | 7.23E-04 | | 5.58E-03 |
|  | **Stress response and inflammation** | | | |  |  | |  |
| hsa04610 | Complement and coagulation cascades | | 69 | | 0.43 | 1.22E-03 | | 7.47E-03 |
| hsa04670 | Leukocyte transendothelial migration | | 115 | | 0.51 | 1.50E-03 | | 8.61E-03 |
| hsa05130 | Pathogenic Escherichia coli infection - EHEC | | 49 | | 0.35 | 1.81E-04 | | 1.56E-03 |
| hsa05020 | Prion diseases | | 36 | | 0.32 | 3.23E-04 | | 2.67E-03 |
| GO:0016477 | Cell migration | | 297 | | 0.35 | 3.72E-18 | | 6.23E-16 |
| GO:0050900 | Leukocyte migration | | 57 | | 0.36 | 1.11E-04 | | 1.15E-03 |
| GO:0030335 | Positive regulation of cell migration | | 60 | | 0.28 | 1.82E-08 | | 5.31E-07 |
| GO:0030336 | Negative regulation of cell migration | | 50 | | 0.31 | 5.45E-06 | | 8.46E-05 |
| GO:0042060 | Wound healing | | 155 | | 0.32 | 5.07E-13 | | 3.52E-11 |
| GO:0032103 | Positive regulation of response to external stimulus | | 46 | | 0.36 | 5.59E-04 | | 4.46E-03 |
| GO:0070887 | Cellular response to chemical stimulus | | 202 | | 0.48 | 4.87E-06 | | 7.66E-05 |
| GO:0004181 | Metallocarboxypeptidase activity | | 25 | | 0.3 | 8.97E-04 | | 6.65E-03 |
| GO:0030030 | Cell projection organization | | 277 | | 0.54 | 1.83E-05 | | 2.45E-04 |
| GO:0009408 | Response to heat | | 33 | | 0.33 | 7.70E-04 | | 5.85E-03 |
| GO:0005126 | Cytokine receptor binding | | 173 | | 0.56 | 1.27E-03 | | 8.80E-03 |
| GO:0006986 | Response to unfolded protein | | 67 | | 0.38 | 1.01E-04 | | 1.05E-03 |
|  | **Neuromuscular junction** | | | | |  | |  |
| hsa04360 | Axon guidance | | 129 | 0.52 | | 1.42E-03 | | 8.44E-03 |
| GO:0022008 | Neurogenesis | | 386 | 0.65 | | 9.21E-04 | | 6.79E-03 |
|  | **Angiogenesis** | | |  | |  | |  |
| GO:0001568 | Blood vessel development | | 225 | 0.31 | | 1.48E-19 | | 2.60E-17 |
| GO:0001935 | Endothelial cell proliferation | | 34 | 0.31 | | 2.48E-04 | | 2.24E-03 |
| GO:0048659 | Smooth muscle cell proliferation | | 28 | 0.3 | | 4.72E-04 | | 3.86E-03 |
| GO:0035239 | Tube morphogenesis | | 70 | 0.43 | | 1.00E-03 | | 7.24E-03 |
| GO:0042310 | Vasoconstriction | | 27 | 0.31 | | 1.02E-03 | | 7.37E-03 |
|  | **Tissue regeneration and remodeling** | | |  | |  | |  |
| hsa05200 | Pathways in cancer | | 327 | 0.53 | | 4.47E-06 | | 7.08E-05 |
| hsa05222 | Small cell lung cancer | | 86 | 0.4 | | 7.27E-05 | | 7.27E-04 |
| GO:0048646 | Anatomical structure formation involved in morphogenesis | | 294 | 0.38 | | 4.13E-15 | | 3.71E-13 |
| GO:0051241 | Negative regulation of multicellular organismal process | | 114 | 0.4 | | 3.99E-06 | | 6.42E-05 |
| GO:0001837 | Epithelial to mesenchymal transition | | 22 | 0.24 | | 2.36E-05 | | 3.02E-04 |
| GO:0050673 | Epithelial cell proliferation | | 50 | 0.33 | | 2.57E-05 | | 3.25E-04 |
| GO:0008544 | Epidermis development | | 151 | 0.41 | | 2.02E-07 | | 4.68E-06 |
| GO:0048762 | Mesenchymal cell differentiation | | 43 | 0.32 | | 7.55E-05 | | 8.15E-04 |
| GO:0010463 | Mesenchymal cell proliferation | | 10 | 0.19 | | 3.61E-04 | | 3.05E-03 |
| GO:0048468 | Cell development | | 593 | 0.56 | | 9.01E-09 | | 2.84E-07 |
| GO:0048771 | Tissue remodeling | | 94 | 0.43 | | 1.23E-04 | | 1.25E-03 |
| GO:0008361 | Regulation of cell size | | 190 | 0.52 | | 1.21E-04 | | 1.24E-03 |
| GO:0007507 | Heart development | | 159 | 0.51 | | 2.09E-04 | | 1.94E-03 |
| GO:0005543 | Phospholipid binding | | 158 | 0.54 | | 8.83E-04 | | 6.58E-03 |
| GO:0046467 | Membrane lipid biosynthetic process | | 31 | 0.33 | | 1.11E-03 | | 7.86E-03 |
| GO:0002062 | Chondrocyte differentiation | | 19 | 0.28 | | 1.42E-03 | | 9.75E-03 |
|  | **Skeletal muscle hypertrophy** | | | | |  | |  |
| GO:0060537 | Muscle tissue development | | 127 | 0.42 | | 5.04E-06 | | 7.87E-05 |
| GO:0010557 | Positive regulation of macromolecule biosynthetic process | | 516 | 0.57 | | 3.09E-07 | | 6.77E-06 |
| GO:0043292 | Contractile fiber | | 105 | 0.47 | | 4.49E-04 | | 3.71E-03 |
| GO:0048747 | Muscle fiber development | | 54 | 0.38 | | 4.56E-04 | | 3.75E-03 |
| GO:0014812 | Muscle cell migration | | 10 | 0.2 | | 5.55E-04 | | 4.43E-03 |
| GO:0033002 | Muscle cell proliferation | | 39 | 0.35 | | 8.93E-04 | | 6.64E-03 |
|  | **Bone remodeling** | | |  | |  | |  |
| GO:0001501 | Skeletal system development | | 285 | 0.42 | | 2.24E-11 | | 1.13E-09 |
| GO:0045667 | Regulation of osteoblast differentiation | | 30 | 0.31 | | 5.03E-04 | | 4.07E-03 |
|  | **Growth factor activity** | | |  | |  | |  |
| GO:0008083 | Growth factor activity | | 144 | 0.53 | | 1.19E-03 | | 8.36E-03 |
| GO:0048009 | Insulin-like growth factor receptor signaling pathway | | 17 | 0.24 | | 2.04E-04 | | 1.91E-03 |
| GO:0007179 | Transforming growth factor beta receptor signaling pathway | | 99 | 0.4 | | 1.21E-05 | | 1.72E-04 |
| GO:0005161 | Platelet-derived growth factor receptor binding | | 10 | 0.19 | | 1.89E-04 | | 1.79E-03 |
| GO:0005158 | Insulin receptor binding | | 23 | 0.29 | | 9.83E-04 | | 7.17E-03 |
| GO:0048545 | Response to steroid hormone stimulus | | 64 | 0.42 | | 1.26E-03 | | 8.79E-03 |
|  | **Blood coagulation** | | |  | |  | |  |
| GO:0007596 | Blood coagulation | | 105 | 0.36 | | 2.85E-07 | | 6.36E-06 |
| GO:0008201 | Heparin binding | | 89 | 0.35 | | 4.03E-07 | | 8.43E-06 |
| GO:0031091 | Platelet alpha granule | | 45 | 0.3 | | 1.25E-05 | | 1.76E-04 |
| GO:0042730 | Fibrinolysis | | 17 | 0.23 | | 1.08E-04 | | 1.12E-03 |
|  | **Cell development** | | |  | |  | |  |
| GO:0045165 | Cell fate commitment | | 101 | 0.46 | | 3.68E-04 | | 3.11E-03 |
| GO:0016049 | Cell growth | | 178 | 0.54 | | 3.93E-04 | | 3.31E-03 |
| GO:0000902 | Cell morphogenesis | | 294 | 0.53 | | 2.85E-06 | | 4.76E-05 |
| GO:0045596 | Negative regulation of cell differentiation | | 145 | 0.5 | | 2.69E-04 | | 2.41E-03 |
| GO:0008284 | Positive regulation of cell proliferation | | 309 | 0.45 | | 4.98E-10 | | 1.95E-08 |
| GO:0006929 | Substrate-bound cell migration | | 11 | 0.17 | | 1.36E-05 | | 1.90E-04 |
|  | **Transcription and translation** | | | | |  | |  |
| GO:0045893 | Positive regulation of transcription, DNA-dependent | | 374 | 0.58 | | 1.57E-05 | | 2.16E-04 |
| GO:0016564 | Transcription repressor activity | | 262 | 0.6 | | 8.55E-04 | | 6.44E-03 |
| GO:0003714 | Transcription corepressor activity | | 128 | 0.51 | | 8.98E-04 | | 6.65E-03 |
|  | **Cell death** | |  |  | |  | |  |
| GO:0043069 | Negative regulation of programmed cell death | | 293 | 0.61 | | 6.11E-04 | | 4.82E-03 |
|  | **Carbohydrate metabolism** | | | | |  | |  |
| GO:0010906 | Regulation of glucose metabolic process | | 22 | 0.28 | | 7.33E-04 | | 5.65E-03 |
|  | **Cell communication** | | |  | |  | |  |
| GO:0005901 | Caveola | | 29 | 0.32 | | 8.60E-04 | | 6.46E-03 |
| GO:0010647 | Positive regulation of cell communication | | 255 | 0.6 | | 9.99E-04 | | 7.24E-03 |
| **Concepts enriched with down-regulated genes** | | | | | | | | |
|  | **Mitochondrial Part and Oxidative phosphorylation** | | |  | | |  |  |
| hsa00190 | Oxidative phosphorylation | | 116 | 3.86 | | | 6.13E-08 | 2.33E-06 |
| hsa00071 | Fatty acid metabolism | | 41 | 4.68 | | | 4.99E-05 | 5.30E-04 |
| GO:0044429 | Mitochondrial part | | 528 | 2.84 | | | 8.34E-18 | 1.23E-15 |
| GO:0006091 | Generation of precursor metabolites and energy | | 305 | 1.72 | | | 8.77E-04 | 6.55E-03 |
| GO:0005761 | Mitochondrial ribosome | | 46 | 8.43 | | | 3.12E-12 | 1.88E-10 |
| GO:0006120 | Mitochondrial electron transport, NADH to ubiquinone | | 36 | 7.41 | | | 1.28E-08 | 3.96E-07 |
| GO:0051536 | Iron-sulfur cluster binding | | 46 | 3.38 | | | 1.33E-03 | 9.21E-03 |
| GO:0018130 | Heterocycle biosynthetic process | | 42 | 3.96 | | | 3.34E-04 | 2.88E-03 |
| GO:0006635 | Fatty acid beta-oxidation | | 27 | 4.91 | | | 4.19E-04 | 3.50E-03 |
| GO:0009055 | Electron carrier activity | | 202 | 1.99 | | | 4.80E-04 | 3.91E-03 |
| GO:0005747 | Mitochondrial respiratory chain complex I | | 36 | 7.34 | | | 1.66E-08 | 4.89E-07 |
| GO:0045333 | Cellular respiration | | 85 | 3.13 | | | 6.34E-05 | 7.10E-04 |
| GO:0042375 | Quinone cofactor metabolic process | | 11 | 7.76 | | | 7.64E-04 | 5.83E-03 |
| GO:0050662 | Coenzyme binding | | 161 | 2.61 | | | 7.89E-06 | 1.20E-04 |
|  | **Peroxisome** | | |  | | |  |  |
| GO:0007031 | Peroxisome organization | | 21 | 5.99 | | | 2.06E-04 | 1.92E-03 |
| GO:0042579 | Microbody | | 98 | 3.54 | | | 1.12E-06 | 2.07E-05 |
|  | **Proteolysis** | | |  | | |  |  |
| hsa00280 | Valine, leucine and isoleucine degradation | | 43 | 5.83 | | | 1.01E-06 | 2.13E-05 |
| GO:0051438 | Regulation of ubiquitin-protein ligase activity | | 75 | 3.3 | | | 6.86E-05 | 7.60E-04 |
|  | **Disease related** | | |  | | |  |  |
| hsa05016 | Huntington's disease | | 170 | 2.88 | | | 8.81E-07 | 2.09E-05 |
| hsa05012 | Parkinson's disease | | 113 | 3.11 | | | 9.74E-06 | 1.40E-04 |
|  | **Carbohydrate metabolism** | | | | | |  |  |
| hsa00640 | Propanoate metabolism | | 32 | 5.97 | | | 1.02E-05 | 1.40E-04 |
| hsa00650 | Butanoate metabolism | | 33 | 4.14 | | | 9.36E-04 | 5.93E-03 |
|  | **Transcription and translation** | | | | | |  |  |
| hsa03420 | Nucleotide excision repair | | 44 | 4.89 | | | 1.48E-05 | 1.90E-04 |
| hsa03022 | Basal transcription factors | | 34 | 5.31 | | | 3.49E-05 | 4.10E-04 |
| hsa03020 | RNA polymerase | | 28 | 5.55 | | | 7.97E-05 | 7.60E-04 |
| hsa03430 | Mismatch repair | | 23 | 5.31 | | | 4.68E-04 | 3.56E-03 |
| hsa03018 | RNA degradation | | 56 | 3.32 | | | 6.18E-04 | 4.51E-03 |
| hsa03030 | DNA replication | | 36 | 4.1 | | | 6.51E-04 | 4.58E-03 |
| hsa00240 | Pyrimidine metabolism | | 94 | 2.6 | | | 7.74E-04 | 5.07E-03 |
| GO:0022613 | Ribonucleoprotein complex biogenesis | | 170 | 2.28 | | | 1.00E-04 | 1.05E-03 |
| GO:0006396 | RNA processing | | 525 | 2.3 | | | 1.55E-11 | 8.02E-10 |
| GO:0006368 | RNA elongation from RNA polymerase II promoter | | 46 | 6.49 | | | 8.71E-09 | 2.76E-07 |
| GO:0003735 | Structural constituent of ribosome | | 150 | 3.36 | | | 1.46E-08 | 4.44E-07 |
| GO:0003899 | DNA-directed RNA polymerase activity | | 40 | 4.23 | | | 1.90E-04 | 1.80E-03 |
| GO:0008168 | Methyltransferase activity | | 163 | 2.71 | | | 2.67E-06 | 4.54E-05 |
| GO:0000123 | Histone acetyltransferase complex | | 50 | 4.2 | | | 3.60E-05 | 4.30E-04 |
| GO:0016410 | N-acyltransferase activity | | 82 | 3 | | | 1.73E-04 | 1.67E-03 |
| GO:0006367 | Transcription initiation from RNA polymerase II promoter | | 71 | 3.87 | | | 5.73E-06 | 8.79E-05 |
| GO:0006412 | Translation | | 379 | 1.92 | | | 7.07E-06 | 1.10E-04 |
| GO:0004526 | Ribonuclease P activity | | 10 | 7.92 | | | 1.09E-03 | 7.73E-03 |
| GO:0042175 | Nuclear envelope-endoplasmic reticulum network | | 216 | 1.87 | | | 1.03E-03 | 7.42E-03 |
| GO:0006297 | Nucleotide-excision repair, DNA gap filling | | 17 | 6.24 | | | 5.23E-04 | 4.20E-03 |
| GO:0006325 | Chromatin organization | | 353 | 1.72 | | | 3.42E-04 | 2.94E-03 |
|  | **Oxygen transport** | | |  | | |  |  |
| GO:0005833 | Hemoglobin complex | | 12 | 1.04 | | | 1.11E-05 | 1.60E-04 |
|  | **Protein modification** | | |  | | |  |  |
| GO:0008430 | Selenium binding | | 27 | 5.38 | | | 1.31E-04 | 1.32E-03 |
| GO:0005789 | Endoplasmic reticulum membrane | | 202 | 2.04 | | | 2.83E-04 | 2.50E-03 |
| GO:0006497 | Protein amino acid lipidation | | 51 | 3.31 | | | 9.42E-04 | 6.92E-03 |
| GO:0016272 | Prefolding complex | | 10 | 9.08 | | | 2.72E-04 | 2.42E-03 |
