# Supplementary material for: Skeletal muscle gene expression in response to resistance exercise: sex specific regulation
Source: BMC Genomics. 2010 Nov 24;11:659. doi: 10.1186/1471-2164-11-659 (PMC3091777; doi:10.1186/1471-2164-11-659)
Supplement: Additional file 5 — Table S5: Enriched biological concepts for up- and down-regulated genes in female biceps 24 h post-RE. [file 1471-2164-11-659-S5.DOCX]

| **Table S5. Enriched biological concepts for up- and down-regulated genes in female biceps 24h post-RE.** | | | | | |
| --- | --- | --- | --- | --- | --- |
| KEGG pathways and GO terms having *FDR<0.01* from LRpath analysis are shown (redundant GO terms were collapsed based on substantial overlap of genes and/or parent-child relationship between relevant GO terms). *Odds ratios* were calculated based on the difference between a *p-value*=0.50 and a *p-value*=0.001; *gene #* indicates how many analyzed genes belong to each enriched category; *p Value* indicates significance of enrichment testing by LRpath analysis; *FDR*, False Discovery Rates, significance statistic adjusted for multiple testing. | | | | | |
|  |  |  |  |  |  |
| Concept ID | Concept Name | Gene # | Odds Ratio | p Value | FDR |
| **Concepts enriched with up-regulated genes** | | | |  |  |
|  | **Gene translation and protein biosynthesis** | |  |  |  |
| hsa00970 | Aminoacyl-tRNA biosynthesis | 41 | 0.06 | 1.51E-05 | 2.24E-03 |
| GO:0006412 | Translation | 379 | 0.24 | 4.93E-08 | 3.03E-05 |
| GO:0005783 | Endoplasmic reticulum | 886 | 0.39 | 1.75E-07 | 8.07E-05 |
| GO:0044431 | Golgi apparatus part | 258 | 0.29 | 1.31E-04 | 9.66E-03 |
| GO:0009119 | Ribonucleoside metabolic process | 41 | 0.04 | 3.74E-06 | 1.06E-03 |
|  | **Mitochondrial biogenesis** | | |  |  |
| GO:0005761 | Mitochondrial ribosome | 46 | 0.07 | 4.48E-05 | 4.34E-03 |
|  | **Others** |  |  |  |  |
| hsa04540 | Gap junction | 89 | 0.12 | 2.36E-05 | 2.24E-03 |
| hsa05130 | Pathogenic Escherichia coli infection - EHEC | 49 | 0.09 | 1.45E-04 | 8.68E-03 |
| GO:0005525 | GTP binding | 347 | 0.31 | 2.99E-05 | 3.33E-03 |
| GO:0003924 | GTPase activity | 187 | 0.24 | 1.45E-04 | 9.87E-03 |
| **Concepts enriched with down-regulated genes** | | | | |  |
| GO:0005833 | Hemoglobin complex | 12 | 397.27 | 8.16E-11 | 3.00E-07 |
| GO:0005863 | Striated muscle thick filament | 17 | 82.48 | 2.97E-06 | 9.95E-04 |
| GO:0003700 | Transcription factor activity | 893 | 2.1 | 8.57E-05 | 7.33E-03 |
| GO:0002376 | Immune system process | 934 | 2.11 | 5.24E-05 | 4.94E-03 |
