# Supplementary material for: Skeletal muscle gene expression in response to resistance exercise: sex specific regulation
Source: BMC Genomics. 2010 Nov 24;11:659. doi: 10.1186/1471-2164-11-659 (PMC3091777; doi:10.1186/1471-2164-11-659)
Supplement: Additional file 6 — Table S6: Differential expression of the selected genes in exercised vs. rested muscle measured by qRT-PCR. [file 1471-2164-11-659-S6.DOCX]

| **Table S6. Differential expression of the selected genes in exercised vs. rested muscle.** | | | | | | | | | | | |  |
| --- | --- | --- | --- | --- | --- | --- | --- | --- | --- | --- | --- | --- |
| Gene Symbol | Male 4h | |  | Male 24h | |  | Female 4h | |  | Female 24h | | |
|  | Microarray | RT-PCR |  | Microarray | RT-PCR |  | Microarray | RT-PCR |  | Microarray | RT-PCR | |
| FBXO40 | -0.39 | 0.16 |  | -0.56 | 0.09 |  | -0.14 | -0.12 |  | 0 | -0.56 | |
| VEGFA | 0.45 | 1.41 |  | 0.19 | 0.97 |  | 1.26 | 2.22 |  | 0.15 | -0.03 | |
| KDR | 0.65 | 0.96 |  | 0.93 | 1.95 |  | 0.99 | 2.05 |  | 0.21 | -0.26 | |
| ALDH2 | -0.39 | -1.02 |  | -0.80 | -0.28 |  | -0.55 | -0.60 |  | -0.15 | -1.54 | |
| IGF1 | 0.33 | 0.67 |  | 1.14 | 1.46 |  | 0.98 | 1.54 |  | 0.48 | 0.62 | |
| PFKFB3 | -0.06 | 0.83 |  | -2.62 | -3.85 |  | 0.32 | 2.07 |  | -0.59 | -2.31 | |
| IRS2 | 0.44 | 3.19 |  | -0.29 | -0.58 |  | 1.39 | 2.68 |  | -0.28 | -1.81 | |
| SMAD3 | 0.30 | 0.83 |  | -0.23 |  |  | 0.63 | 1.44 |  | -0.21 | -0.88 | |
| PPARGC1 | 0.73 | 2.20 |  | -0.06 | 0.98 |  | 0.31 | 3.19 |  | 0 | 0.44 | |
| DUSP1 | 0.08 | 1.31 |  | -0.43 | -0.05 |  | 0.58 | 1.61 |  | -0.32 | -1.27 | |
| Numbers are fold changes as log _2_ (exercised/rested). | | | | | | | | | | | |  |
