# Supplementary material for: Skeletal muscle gene expression in response to resistance exercise: sex specific regulation
Source: BMC Genomics. 2010 Nov 24;11:659. doi: 10.1186/1471-2164-11-659 (PMC3091777; doi:10.1186/1471-2164-11-659)
Supplement: Additional file 8 — Discussion on common GO terms and KEGGs pathways regulated by RE in both male and female biceps. [file 1471-2164-11-659-S8.DOCX]

# Skeletal muscle gene expression in response to resistance exercise: sex specific regulation

### Dongmei Liu^1^, Maureen A. Sartor^2^, Gustavo A. Nader^3^, Laurie Gutmann^4^, Mary Kay Treutelaar^5^, Emidio E. Pistilli^6^, Heidi B. IglayReger^1^, Charles F. Burant^5^, Eric P. Hoffman^7^, and Paul M. Gordon^1^*

1 Laboratory for Physical Activity and Exercise Intervention Research, Dept. of Physical Medicine and Rehabilitation, University of Michigan, Ann Arbor, MI

2 Center for Computational Medicine and Biology, University of Michigan, Ann Arbor, MI, USA.

3 Department of Medicine, Karolinski Institute, Stockholm, Sweden

4 Dept of Neurology, West Virginia University, Morgantown, WV

^5^ Dept. of Internal Medicine^,^ University of Michigan, Ann Arbor, MI

^6^ Department of Physiology and Pennsylvania Muscle Institute, University of Pennsylvania, Philadelphia, PA

7 Research Center for Genetic Medicine, Children’s National Medical Center, Washington, DC

*Corresponding author

Email addresses: DL: dongmei@med.umich.edu

MAS: sartorma@umich.edu

GAN: gustavo.nader@ki.se

LG: lgutmann@hsc.wvu.edu

MAT: mtreut@med.umich.edu

EEP: epist@mail.med.upenn.edu

HBI: iglay@med.umich.edu

CFB: burantc@med.umich.edu

EPH: ehoffman@cnmcresearch.org

PMG: gordonp@med.umich.edu

## Common biological processes transcriptionally up-regulated by RE

***Extracellular matrix and actin cytoskeleton***. The most pronounced and consistent finding included up-regulation of genes involved in construction and organization of extracellular matrix (ECM) and actin cytoskeleton, as well as a myriad of biological processes based on these molecular structures such as cell migration and integrin-mediated signalling. The implication of the ECM-actin cytoskeleton system in mediating skeletal muscle responses and adaptation to mechanical loading has been documented [1,2]. During muscle contraction or stretching, the ECM receptor interaction has been implicated in coupling of mechanical stimuli with intracellular biochemical events leading to protein synthesis or degradation [3]. Activation of cellular signalling through the ECM-integrin interactions, including formation of focal adhesion complexes, has been suggested to be the earliest and most critical component for transduction of mechanical signalling and focal adhesion complexes [1]. This may also serve as a key integration site for multiple signals that are present during overload-induced skeletal muscle hypertrophy [1]. Disruption of ECM experimentally prohibited terminal differentiation of myoblasts [4], whereas overproduction of integrin protected skeletal muscle injury during downhill running exercise in transgenic mice [5]. Giannelli et al. [6] suggested that matrix metalloproteinase (*MMP*) imbalance may play a role in muscle disuse atrophy. In the present study, along with various ECM components and integrin subunits, metallopeptidase inhibitors (*TIMPs*), including *TIMP1*, *TIMP2* and *TIMP3*, were consistently up-regulated after exercise. Our data support the notion that the ECM-integrin-cytoskeletal system plays an important role in mediating RE-induced muscle adaptation.

***RE induced tissue remodelling****.* Induction of tissue development in both male and female skeletal muscle following RE was observed. Such concepts included cell proliferation, cellular component morphogenesis and muscle tissue development. We observed several significant genes involved in multiple enrichment concepts and are likely to be the key genes driving the concomitant enrichment of the relevant concepts. These genes included xin actin-binding repeat containing 1 (*XIRP1*), integrin beta 1 binding protein 3 (*ITGB1BP3*), insulin-like growth factor 1 (*IGF1*), actin, alpha, cardiac muscle 1 (*ACTC1*), and caveolin 1 and 2 (*CAV1, CAV2*). Although muscle biopsy is comprised primarily of muscle fibres, other cell types also exist including fibroblasts, endothelial cells, smooth muscle cells, neurons and blood cells, and they could respond to mechanical strain induced by muscle contraction by altering their transcriptional profiles. The up-regulation of genes involved in nervous system development, bone remodelling and angiogenesis detected among our subjects may represent evidence for the cross-talk between muscle fibres and their surrounding connective tissues and the cells with which it is in direct contact [7]. It is easy to perceive that increases in the size of muscle fibres would only be fully functional if there were simultaneously coordinated events indicating adaptation and remodelling in the supporting structures and cells comprising the muscle tissue.

***RE induced angiogenesis****.* We observed that genes involved in angiogenesis showed augmented expression at early recovery time points in both males and females. A proportional increase of capillary density (per muscle fiber) has been reported to accompany muscle hypertrophy after RE training [8]. In agreement with previous studies [9,10], we observed consistent up-regulation of key regulators of angiogenesis, especially at 4h post-exercise. These included vascular endothelial growth factor A (*VEGFA*), kinase insert domain receptor (*KDR*), fms-related tyrosine kinase 1 (*FLT1*), neuropilin 1 (*NRP1*), hypoxia-inducible factor 1, alpha subunit (basic helix-loop-helix transcription factor) (*HIF1A*), angiopoietin 1 (*ANGPT1*), angiopoietin 2 (*ANGPT2*), tyrosine kinase, endothelial (venous malformations, multiple cutaneous and mucosal) (*TEK*), and cysteine-rich angiogenic protein 61 (*Cyr61*).

***RE augmented signal transduction****.* Our data also portrayed a complex signalling transduction network implicated in skeletal muscle transcriptional regulation in response to RE. Up-regulated signalling pathways included G-protein coupled receptor protein signalling, calcium binding, small GTPase mediated signal transduction, integrin-mediated signalling, and cAMP mediation signalling, as well as MAPK signalling pathway. The important role of each signalling pathways in mediating muscle hypertrophy has been documented [1], though substantially more attention has been focused on the post-translational modification of the signalling cascades. The findings from the present study indicate that transcriptional regulations of factors involved in each signalling pathway were also involved in response to RE, and these factors are more likely to mediate muscle adaptation to chronic training since the post-translational activation such as phosphorylation only lasts for a few hours after exercise. Our data indicate that transcriptional induction of MAPK kinases, and MAP kinase phosphatase, both dual specificity phosphatases, are involved in MAPK activation in skeletal muscle RE response.

***RE elevated expression of stress response genes****.* Early activation of the immune system following RE has also been manifested in our data by showing up-regulation of genes involved in leukocyte chemotaxis and transendothelial migration, and wound healing, which was again consistent between sexes. This is not surprising considering that micro damages to myofiber are very likely to happen in an exercise program involving an eccentric lengthening component and a relatively high volume as was the case with our exercise protocol. Moreover, it has been suggested that the inflammatory response is integral to muscle growth induced by resistance exercise involving eccentric muscle contractions [11]. Most of these processes, along with ones related to cell-cell communication such as adherens junction were activated early during recovery and returned to basal levels by 24h post exercise. However, in contrast to other studies [12], we did not see elevated expression of cytokine genes such as *IL1*, *IL6*, *IL8*, *IL15*, and *TNF*, though *IL6R* was up-regulated. Along with immune system activation, genes involved in the stress responses were up-regulated and the anti-oxidant system was down-regulated including members of antioxidant metallothioneins (i.e., *MT1H*, *MT1M*, *MT1X,* *MT1F* and *MT1G*). Similar results have been documented in animal studies [13].

## Common biological processes transcriptionally down-regulated by RE

***RE repressed mitochondrial structural and functional factors****.* Consistent across sexes, we observed significant enrichment of biological processes relevant to muscle protein catabolism, mitochondrial part and energy derivation by oxidation of organic compounds, fatty acid oxidation, and carbohydrate metabolism. Resistance exercise training has been shown to decrease expression of genes related to mitochondrial/oxidative capacity [14]. As discussed above, genes related to lipid metabolism and mitochondrial oxidative phophorylation were expressed at higher levels in female muscle in the resting state. A similar response across both sexes to RE were observed with coordinated down-regulation, including *LPL*, *ALDH2*, *SREBF1*, *GPAM*, *ALDH1A1*, *CTP1B*, *PPARA*, *SLC27A1*, and *SLC25A34*. Some metabolic genes showed up-regulation at least at one time point of the recovery period, which might mediate the documented metabolic benefits of RE in muscle such as insulin sensitivity. These genes included *PDK4*, *PPARD*, *PPARGC1*, *UCP3*, *UCP2*, *RRAD*, *DOK7*, *INSIG1*, and *HBEGF*.

***RE repressed expression of proteolytic factors****.* Consistent across sexes, we observed significant enrichment of biological processes relevant to muscle protein catabolism. Direct evidence for suppression of muscle protein break-down following resistance exercise has been rare. However down-regulation of the ubiquitin-proteasome degradation pathway as a consequence of RE has been observed previously [12,15]. Similarly, our results demonstrate that expression of genes encoding subunits of proteasome complex and ubiquitin ligase, including F-box protein 32 (*FBXO32*) and tripartite motif-containing 63 (*TRIM63*), were down-regulated [12]. What’s more, the key regulators functioning in muscle atrophy such as myostatin (*MSTN*), forkhead box O3 (*FOXO3*), SIX homeobox 1 (*SIX1*) were also consistently down-regulated, which is in agreement with Louis et al’s report [12]. However, unexpectedly, we saw up-regulation rather than down-regulation of calpain 2, (m/II) large subunit (*CAPN2*) in both male and female exercised muscles. The present study supports the notion that muscle protein break-down suppression has a great contribution to muscle protein accumulation in RE-induced muscle hypertrophy. This has been not yet been fully studied and warrants further investigation.

***RE repressed expression of protein synthetic regulators.*** Studies on muscle protein synthesis have indicated that muscle protein synthesis decreased during and immediately after exercise, and then increased gradually with a peak increase reached after 24 h post-exercise [16]. This protein synthetic response is thought to be mediated by enhanced phosphorylation of Akt-mTOR-p70S6k [16]. Our expression data suggested the existence of transcriptional regulation relevant to muscle protein synthesis following RE. We observed that gene transcriptions, post-transcriptional processing and mRNA translation were down-regulated at 4h post for both males and females and 24h post exercise for males only. Interestingly, at 24h post in females only, we observed a coordinated up-regulation of protein biosynthesis systems including aminoacyl-tRNA biosynthesis (hsa00970, GO:0006418), translation (GO:0006412), ribonucleoside metabolic process (GO:0009119), endoplasmic reticulum (GO:0005783) and Golgi apparatus part (GO:0044431). Given the delayed recovery response in male muscle, it is reasonable to speculate that these responses might increase above baseline later than 24h post.

Collectively, our data suggest that resistance exercise-induced muscle hypertrophy may occur at the transcriptional level through a decline of protein degradation and stabilization of mRNA at an early time point and augmented translation at a later time point. Intriguingly, among mTOR signalling-associated factors, we found coordinated repression of negative regulators of mTOR only in male muscle 24h. These genes included DNA-damage-inducible transcript 4 (*DDIT4*), DNA-damage-inducible transcript 4-like (*DDIT4L*), AKT1 substrate 1 (proline-rich) (*AKT1S1*), and tuberous sclerosis 1 and 2 (*TSC1*, *TSC2*). This finding may implicate a mechanism behind disproportional muscle growth in males vs. females despite training at a similar relative intensity. Further research in this area is clearly needed.

Reference List

1. Burkholder TJ: **Mechanotransduction in skeletal muscle
63.** *Front Biosci* 2007, **12:** 174-191.

2. Kjaer M: **Role of extracellular matrix in adaptation of tendon and skeletal muscle to mechanical loading
41.** *Physiol Rev* 2004, **84:** 649-698.

3. Hornberger TA, Esser KA: **Mechanotransduction and the regulation of protein synthesis in skeletal muscle
3.** *Proc Nutr Soc* 2004, **63:** 331-335.

4. Osses N, Brandan E: **ECM is required for skeletal muscle differentiation independently of muscle regulatory factor expression
4.** *Am J Physiol Cell Physiol* 2002, **282:** C383-C394.

5. Boppart MD, Burkin DJ, Kaufman SJ: **Alpha7beta1-integrin regulates mechanotransduction and prevents skeletal muscle injury.** *Am J Physiol Cell Physiol* 2006, **290:** C1660-C1665.

6. Giannelli G, De MA, Marinosci F, Antonaci S: **Matrix metalloproteinase imbalance in muscle disuse atrophy
2.** *Histol Histopathol* 2005, **20:** 99-106.

7. Mackey AL, Heinemeier KM, Koskinen SO, Kjaer M: **Dynamic adaptation of tendon and muscle connective tissue to mechanical loading
8.** *Connect Tissue Res* 2008, **49:** 165-168.

8. McCall GE, Byrnes WC, Dickinson A, Pattany PM, Fleck SJ: **Muscle fiber hypertrophy, hyperplasia, and capillary density in college men after resistance training.** *J Appl Physiol* 1996, **81:** 2004-2012.

9. Gavin TP, Drew JL, Kubik CJ, Pofahl WE, Hickner RC: **Acute resistance exercise increases skeletal muscle angiogenic growth factor expression
1.** *Acta Physiol (Oxf)* 2007, **191:** 139-146.

10. Kivela R, Kyrolainen H, Selanne H, Komi PV, Kainulainen H, Vihko V: **A single bout of exercise with high mechanical loading induces the expression of Cyr61/CCN1 and CTGF/CCN2 in human skeletal muscle
1.** *J Appl Physiol* 2007, **103:** 1395-1401.

11. Peake J, Nosaka K, Suzuki K: **Characterization of inflammatory responses to eccentric exercise in humans
2.** *Exerc Immunol Rev* 2005, **11:** 64-85.

12. Louis E, Raue U, Yang Y, Jemiolo B, Trappe S: **Time course of proteolytic, cytokine, and myostatin gene expression after acute exercise in human skeletal muscle
1.** *J Appl Physiol* 2007, **103:** 1744-1751.

13. McGivney BA, Eivers SS, MacHugh DE, MacLeod JN, O'Gorman GM, Park SD *et al*.: **Transcriptional adaptations following exercise in thoroughbred horse skeletal muscle highlights molecular mechanisms that lead to muscle hypertrophy
1.** *BMC Genomics* 2009, **10:** 638.

14. Stepto NK, Coffey VG, Carey AL, Ponnampalam AP, Canny BJ, Powell D *et al*.: **Global gene expression in skeletal muscle from well-trained strength and endurance athletes
1.** *Med Sci Sports Exerc* 2009, **41:** 546-565.

15. Nedergaard A, Vissing K, Overgaard K, Kjaer M, Schjerling P: **Expression patterns of atrogenic and ubiquitin proteasome component genes with exercise: effect of different loading patterns and repeated exercise bouts
4.** *J Appl Physiol* 2007, **103:** 1513-1522.

16. Kumar V, Atherton P, Smith K, Rennie MJ: **Human muscle protein synthesis and breakdown during and after exercise
1.** *J Appl Physiol* 2009, **106:** 2026-2039.
