# Supplementary material for: Skeletal muscle gene expression in response to resistance exercise: sex specific regulation
Source: BMC Genomics. 2010 Nov 24;11:659. doi: 10.1186/1471-2164-11-659 (PMC3091777; doi:10.1186/1471-2164-11-659)
Supplement: Additional file 9 — Table S8: Genes for qRT-PCR validation and TaqMan assay used. [file 1471-2164-11-659-S9.DOCX]

| **Table S8. Genes for qRT-PCR validation and TaqMan assay used.** | | |
| --- | --- | --- |
| Gene Symbol | Gene Name | Assay ID # |
| FBXO40 | F-box protein 40 | Hs00212488_m1 |
| VEGFA | vascular endothelial growth factor A | Hs00900055_m1 |
| KDR | kinase insert domain receptor (a type III receptor tyrosine kinase) | Hs00911700_m1 |
| ALDH2 | aldehyde dehydrogenase 2 family (mitochondrial) | Hs00355914_m1 |
| IGF1 | insulin-like growth factor 1 (somatomedin C) | Hs01547656_m1 |
| PFKFB3 | 6-phosphofructo-2-kinase/fructose-2,6-biphosphatase 3 | Hs00190079_m1 |
| IRS2 | insulin receptor substrate 2 | Hs00275843_s1 |
| B2M | beta-2-microglobulin | Hs00187842_m1 |
| SMAD3 | SMAD family member 3 | Hs00232219_m1 |
| PPARGC1 | peroxisome proliferator-activated receptor gamma, coactivator 1 alpha | Hs01016724_m1 |
| DUSP1 | dual specificity phosphatase 1 | Hs00610257_g1 |
